# A short translational ramp determines the efficiency of protein synthesis

**DOI:** 10.1038/s41467-019-13810-1

**Published:** 2019-12-18

**Authors:** Manasvi Verma, Junhong Choi, Kyle A. Cottrell, Zeno Lavagnino, Erica N. Thomas, Slavica Pavlovic-Djuranovic, Pawel Szczesny, David W. Piston, Hani S. Zaher, Joseph D. Puglisi, Sergej Djuranovic

**Affiliations:** 1 Department of Cell Biology and Physiology, Washington University School of Medicine, 600 South Euclid Avenue, Campus Box 8228, St. Louis, MO 63110 USA; 2 Department of Structural Biology, Stanford University School of Medicine, Stanford, CA 94305-5126 USA; 3 Department of Applied Physics, Stanford University, Stanford, CA 94305-5126 USA; 4 Department of Biology, Washington University, St Louis, MO 63105 USA; 5 Department of Bioinformatics, Institute of Biochemistry and Biophysics Polish Academy of Sciences, Warsaw, Poland; 6 Present Address: Department of Genome Sciences, University of Washington, Seattle, WA USA; 7 Present Address: Experimental Imaging Center, IRCCS Ospedale San Raffaele, Milan, Italy

**Keywords:** RNA, Single-molecule biophysics, Expression systems, Translation

## Abstract

Translation initiation is a major rate-limiting step for protein synthesis. However, recent studies strongly suggest that the efficiency of protein synthesis is additionally regulated by multiple factors that impact the elongation phase. To assess the influence of early elongation on protein synthesis, we employed a library of more than 250,000 reporters combined with in vitro and in vivo protein expression assays. Here we report that the identity of the amino acids encoded by codons 3 to 5 impact protein yield. This effect is independent of tRNA abundance, translation initiation efficiency, or overall mRNA structure. Single-molecule measurements of translation kinetics revealed pausing of the ribosome and aborted protein synthesis on codons 4 and 5 of distinct amino acid and nucleotide compositions. Finally, introduction of preferred sequence motifs only at specific codon positions improves protein synthesis efficiency for recombinant proteins. Collectively, our data underscore the critical role of early elongation events in translational control of gene expression.

## Abstract (web summary)

Several factors contribute to the efficiency of protein expression. Here the authors show that the identity of amino acids encoded by codons at position 3–5 significantly impact translation efficiency and protein expression levels.

## Introduction

The efficiency of protein synthesis is governed by the rates of translation initiation, elongation and to a lesser extent termination^1–5^. Potential factors that contribute to protein synthesis efficiency have been discovered using both endogenous genes and reporter sequences by focusing on tRNA abundance, amino acid sequence or both mRNA sequence and structure^6–19^. Several conflicting models for the efficiency of protein synthesis have been proposed that consider the codon distribution at the N-terminus as well as local mRNA structure around the translation start sites^9,11,14,15,19,20^. Reduced abundance of tRNAs coding for N-terminal protein residues may play a crucial role in slowing down initial rounds of translation elongation^9,14,21^. Such a translational ramp would be beneficial in preventing detrimental collision-dependent abortion of protein synthesis^14,18,22^. Some of these effects can be rationalized by the presence of mRNA structural elements within the first 5–16 codons^11,19,20,23–25^. In addition, interactions between the nascent peptide and the exit tunnel of the ribosome appear to play an important role in dictating peptidyl transfer rates during early elongation events^12,26–28^. However, the mechanism of nucleotide and peptide sequence effects on early elongation and processivity of protein synthesis remain poorly understood. Here, we present data that strongly suggest that the mRNA and the encoded protein sequences of the first five codons are key in dictating the efficiency of protein synthesis.

## Results

### Design of eGFP library and evaluation of protein production

**Fig. 1:**
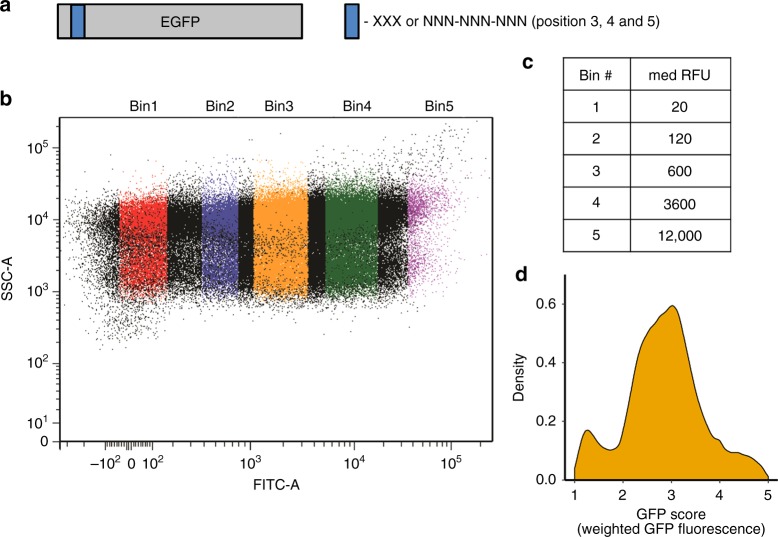
Fluorescence-based screen identifies large differences in protein synthesis as a result of the identity of the N-terminus. **a** Scheme of the reporter system to test the influence of the first five amino acids and mRNA sequence of the first ribosome footprint. Nine random nucleotides were introduced into eGFP reporter in the positions from 7 to 15 nucleotide coding for amino acids 3, 4, and 5 in protein. **b** Fluorescence-activated cell sorting (FACS) of induced *E.coli* cells into 5 bins. Bin 1–4 each represent approximately 24% of the whole cell population depending on eGFP expression. Bin 5 represents 2.5% of the *E.coli* cells with highest eGFP expression based on relative fluorescence values (RFUs). *E.coli* cells were sorted based on granularity (SSC-A) and eGFP fluorescence (FITC-A) channels. **c** Table of relative average fluorescence values for colonies in five separated bins. Wild type eGFP expression is approximately 250 RFUs. **d** Distribution of the plasmid reads based on the GFP score. GFP score represents distribution value for each independent sequence in 5 bins.

To decipher how mRNA sequence and its encoded peptide influence protein synthesis efficiency, we focused on the region surrounding the +10 nucleotide position in an engineered GFP-reporter sequence. This region has been implicated in regulating protein-expression levels by modulating either the efficiency of translation initiation or elongation^11,15,19,20,29^. Studies comparing the first 11 codons of 137 endogenous essential *E. coli* genes^20^ or 756 randomly generated initial 13 codons^29^ pointed to the region around nucleotide +10 (codons 3–5) as important for efficient protein expression. Genome-wide ribosome-profiling studies in yeast and mammalian cells also indicate that translation of the first five amino acids results in ribosomal pausing due to the geometry of the exit tunnel regardless of amino acid sequence^26,30^. To determine the role of amino acid sequence, we created a library of an otherwise codon-optimized eGFP gene with insertion of nine random nucleotides after the second codon (Fig. 1a). Sequencing of the plasmid library revealed 259,134 unique sequences out of the 262,144 possible synthetic eGFP constructs (Supplementary Data 1–5). These were identical except for the 3rd–5th codons (nucleotides 7–15) of the open reading frame. These three codons code for 9261 different tripeptides including truncated peptides due to the presence of one or more stop codons. We used a sort-and-sequence approach to assess the expression of each variant in *E. Coli* (DH5α) (Fig. 1b and Supplementary Fig. 1). Cells were sorted into five bins based on expression of GFP fluorescence which spanned three orders of magnitude (Fig. 1c and Supplementary Fig. 1). The fluorescence variation is larger than previously reported for expression of 14,000 synonymous codon variants of super-folder green fluorescent protein (sfGFP) with randomized promoters, ribosome-binding sites, and the first 11 codons^20^. It is also higher than that reported for 756 constructs with random first 13 codons^29^ and when 94% of the eGFP protein was recoded using synonymous codons^11^. The difference between eGFP variants (Fig. 1b and Supplementary Fig. 1) closely resembles that of a recently reported study on 244,000 synthetic sequences with variance in the first 33 codons assayed in *E*. coli^19^. However, in comparison to these previous studies^11,19,20,26,29,30^ our findings indicate that the overall expression of the protein could be significantly changed as a result of differences in the N-terminal sequence that encompasses amino acids 3–5 of the protein. These amino acids correspond to nucleotides 7–15 of the open-reading frame (ORF) and the first ribosome footprint after initiation (Supplementary Fig. 2).

To assign the level of eGFP expression for each variant, we use a GFP score calculated from the weighted distribution of each independent sequence over five FACS sorted bins (Fig. 1d and Supplementary Figs. 1 and 3). A GFP score close to 1 indicates sequences with low eGFP (median RFU of 50, Fig. 1c and Supplementary Data 1–5); a GFP score of 5 specifies sequences that are highly expressed (median RFU of 12,000, Fig. 1c and Supplementary Data 1–5). While GFP score does not provide linear correlation with eGFP fluorescence, it represents an estimate of the relative expression levels of each eGFP variant in our library. GFP scores were reproducible (sequences with >100 reads) with a Pearson correlation of 0.74 among biological replicates. The average GFP score of the library was ~3, with most of the sequences distributed between bins 2–4 (median RFUs of 120, 600, and 3600, respectively). Since *amber* stop codon (UAG) suppression in DH5α is highly efficient (75–95%)^31^, we used this feature of DH5α cells to compare eGFP variants with *amber* stop codon or other stop codons (*opal*-UGA, *ochre*-UAA, Supplementary Fig. 4). *Amber* suppressor mutation in DH5α cells of tRNA (supE44)^31^ coding for tRNA^Gln^_CUA_ leads to Gln incorporation at UAG codon and served as an additional control for the codon-anticodon interaction and efficiency of protein synthesis (Supplementary Fig. 4). While variants with *ochre* and *opal* stop codons distributed between GFP scores of 1 and 2, distribution of the constructs with an *amber* stop codon followed the distribution of the library without stop codons validating our library fluorescence distribution and phenotype of DH5α cells. Interestingly, the distribution of eGFP variants with *amber* stop codon closely resembled the distribution of variants with CAG codon in the same position (Supplementary Fig. 4). This suggests that codon 3–5 peptide sequence contributes to the amount of protein synthesized together with the corresponding nucleotide sequences.

### Early elongation contributes to variation in protein synthesis

**Fig. 2:**
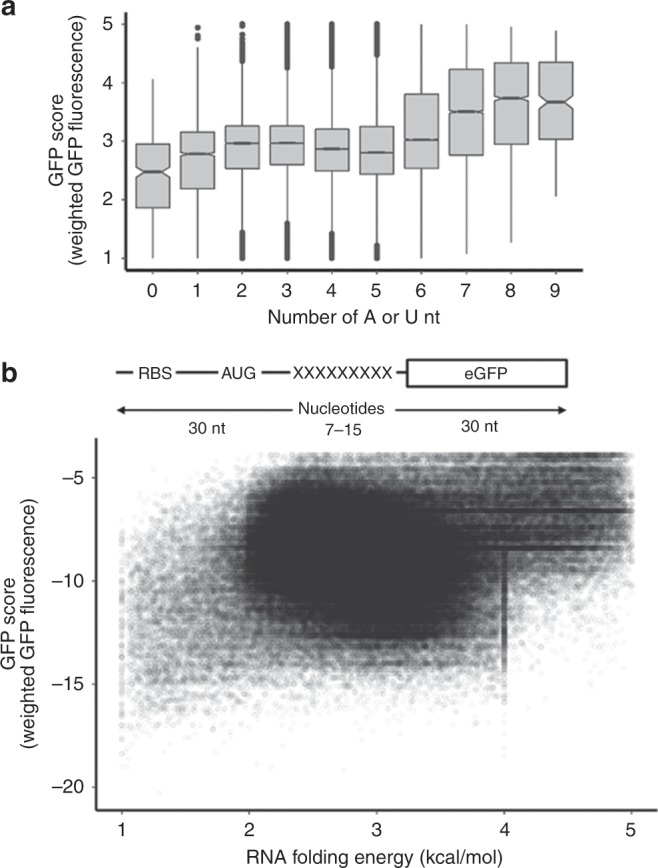
Effect of A-U content and RNA structure on protein expression. **a** Reporters with increased A-U content have slightly higher GFP score. Reporters are binned by the number of A or U nucleotides and plotted against GFP score **b** Influence of local-mRNA structure on expression of eGFP 9nt library. GFP score distribution value is plotted in correlation with the number of A or U nucleotides in 9nt randomized sequence. Boxplot whiskers indicate the furthest datum that is 1.5*Q1 (upper) or 1.5*Q3 (lower).

To test whether eGFP-reporter levels depend on tRNA abundance or rare codons at the start of the coding sequence, we compared the distribution of the GFP scores of all library variants to these features (Supplementary Fig. 5). We did not find any obvious correlation of GFP scores with tRNA abundance, measured by tRNA adaptation index (tAI)^32^, or rare codons (Arg, Ile, or Leu) at codon 3–5 (Supplementary Fig. 5). Amino acid chemical properties such as overall charge or hydrophobicity of the encoded tri-peptides (Supplementary Fig. 6) or plasmid abundance in the unsorted cells (Supplementary Fig. 3) also showed no correlation with GFP score. Instead, we found that GFP score correlated moderately with the AU content of the variable region similar to the so-called downstream box element (Fig. 2a)^19,23,29,33,34^. On average, eGFP variants that harbored 6–9A or U nucleotides at positions +7 to +15 had on average better expression than the rest of the library variants. This was further confirmed with more thorough analysis of the library sequences divided into four categories defined by GFP score (Supplementary Fig. 7). Sequence motif analysis of variants with highest GFP scores (GFP score >4) indicated slight AU bias; however, there was no strong bias against GC rich sequences (Supplementary Fig. 7). Sequences that were moderately expressed had a random distribution of GC nucleotides. Low-expressed sequences have a slight increase in C nucleotides (1 ≤ GFP score < 2). This could potentially be caused by the C-rich proline codons, which are well documented to stall translation^35–37^. Taken together, these analyses indicate that local mRNA sequence and potentially base-pairing stability of nucleotides +7 to +15 influence the expression of proteins.

Given that high AU-content correlated slightly with eGFP expression, we asked whether local mRNA structure, or certain nucleotide or amino acid sequences, were responsible for this correlation. Using RNAfold^38^ we analyzed the variable region (nucleotides 7–15) including ±30 nucleotides around it (Fig. 2b). The majority of RNA secondary structures had folding energies (Δ*G*) ranging from −16 to −4 kcal/mol. On average constructs with poor GFP score (GFP score <2) had slightly stronger RNA folding energies than eGFP constructs with higher (>4) or medium GFP scores (2–4) (Supplementary Fig. 8). Our analyses of RNA folding energy for tested eGFP constructs indicated a rather week correlation between the expression levels of eGFP variants and RNA-folding energy around the start site compared to the recent study looking at variance in the first 33 codons^19^. This is in line with previous observations that differences in the region around the 10th nucleotide were the most correlated with reporter expression levels, even for the subset of constructs with the similar total free energy of mRNA folding across the N-terminal region^20^.

**Fig. 3:**
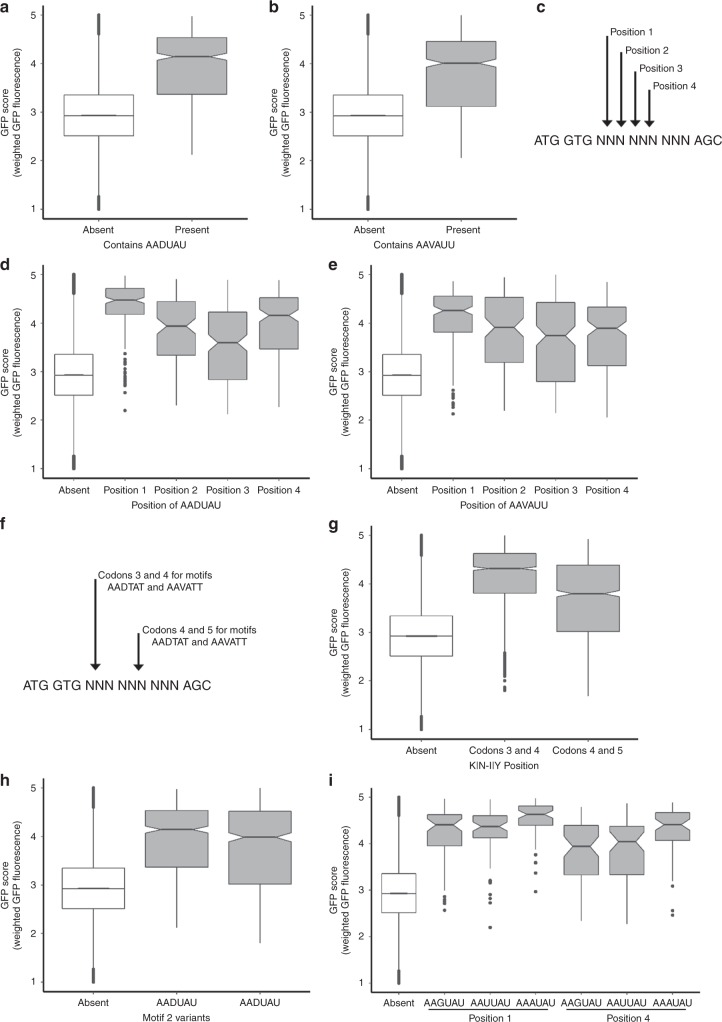
Identification of motifs that correlate with GFP score. **a**, **b** Enrichment analyses of sequenced constructs with average GFP score of ≥4.0 results in two motifs with DNA sequence AADUAU and AAVAUU, or amino acid sequence K|N-Y and K|N-I, respectively. Average GFP score of all sequences with two motifs (present) is compared to the rest of library (absent). **c** Analysis scheme of the GFP scores for two motifs by moving one nucleotide at the time. Position 1 and position 4 code for K|N-Y and K|N-I amino acid motifs as codons 3 and 4 or 4 and 5, respectively. **d**, **e** Analysis of average GFP scores for two sequences motifs based on their position in 9nt randomized sequence indicates potential amino acid dependence. Average GFP score is compared to the rest of library (absent). **f** Scheme of analysis of overall influence of amino acid sequence when motifs code for amino acids in positions 3 and 4 or 4 and 5, respectively. **g** Analysis of overall influence of amino acid sequence of motif K|N-I|Y in positions 3, 4, and 5. Average GFP score for motifs is compared to the rest of library (absent). **h**, **i** Analysis of the influence of degenerate codons for Tyr(Y) or Asn(N) and Lys(K) on the GFP score of AADUAU motif, respectively. All analyzed sequences with stop codons were filtered out to represent average coding library (absent). Comparison is shown vs all the coding constructs in the library. Boxplot whiskers indicate the furthest datum that is 1.5*Q1 (upper) or 1.5*Q3 (lower).

Using a motif-scanning approach, we identified motifs that were enriched in eGFP variants with a score greater than 4, when compared to poorly expressed variants (GFP score < 3). Among several hexanucleotide motifs that were identified, the two most significantly enriched RNA motifs (enrichment ratio of >10 and *p*-value < 1E−5) were AADUAU (D stands for not C, Fig. 3a) and AAVAUU (V stands for not U, Fig. 3b). During decoding, these motifs code for lysine (K) or asparagine (N) and tyrosine (Y) or isoleucine (I), as first and second amino acids, respectively (represented as K|N-Y|I sequence motif). Intriguingly, all eGFP variants with combination of K|N-Y|I amino acids regardless of their synonymous codons had on average a GFP score of 4.31 ± 0.87 (median ± standard deviation) and broad range of RNA-folding energy of Δ*G* = −3.9 kcal/mol to Δ*G* = −12.0 kcal/mol (median Δ*G* = −5.3 kcal/mol). These data argue for possible amino acid or nucleotide contribution for higher expression eGFP variants independently of RNA-folding energies (Supplementary Figs. 9, 10). These same amino acids were identified as occurring more frequently in eGFP variants with high scores (>4) compared to those with low scores (Supplementary Fig. 10). Analyses of the positional bias of hexanucleotide motifs (Fig. 3c) revealed that K|N-Y|I amino acid combination on average had higher GFP scores than any other amino acid combination (Fig. 3d–g). This suggests that besides nucleotide sequence, certain amino acids (K, N, Y, or I) contribute to the expression level of eGFP variants when translated from codons 3–5. We also observed preference for certain amino acids at position 3 when K|N-Y or K|N-I motifs were in position 4 or 5, providing additional support for our model that amino acid sequences contribute to the observed difference in eGFP variants covered by our library (Supplementary Fig. 11). Analysis of the influence of K, N, I, and Y isoacceptor tRNAs indicated small differences between tested codons (Figs. 3e, h and i, Supplementary Fig. 8). A tendency towards low GFP scores was observed for codons with G or C nucleotides and when motifs were shifted to position 4 (Fig. 3h, i, Supplementary Fig. 9). Overall these analyses suggest that both amino acid and nucleotide composition at the N-terminus and beginning of the coding sequence, respectively, contribute to the overall efficiency of protein synthesis.

### Confirmation of library data in vitro and in vivo in *E. coli*

**Fig. 4:**
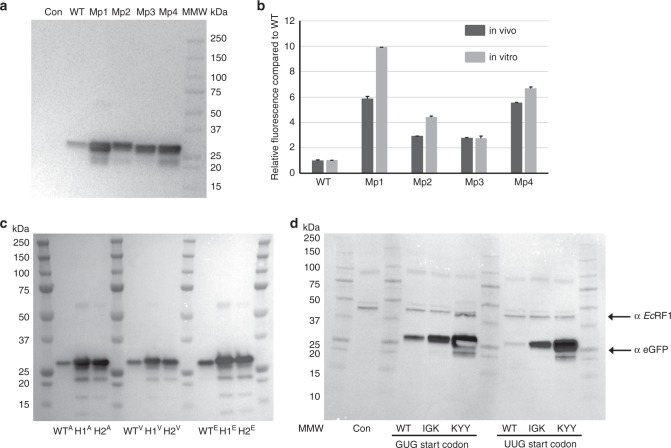
Analysis of the effect of motifs in in vitro and in vivo bacterial expressions assays. **a** Western-blot analysis of NEB Pure Express in vitro expression of eGFP constructs with motif 1 AADUAU in different positions coding for amino acids 3–5. Mp1 indicates motif1 in position 1, Mp2 indicates motif1 in position 2, Mp3 indicates motif1 in position 3 and Mp4 indicates motif1 in position 4, where insertion positions are defined as in Fig. 3c. Wild-type eGFP (WT) control is indicated. Five percent of is analyzed. Kinetics of in vitro translation reaction is shown in Supplementary Fig. 13. **b** Relative eGFP fluorescence from in vitro and in vivo expression of eGFP constructs with AADUAU motif in different position compared to the wild-type eGFP construct. The fluorescence ratio of each construct is plotted as a fold increase over the corresponding WT. Endpoint fluorescence for in vivo induction in *E. coli* cells (Supplementary Fig. 12) and in vitro reaction (in Supplementary Fig. 13) were used to calculate ratios. Error bars represent standard deviation. **c** Western-blot analysis indicates that the N-terminal rule does not influence the expression of eGFP variants from pBAD single copy vector in vivo in *E. coli Top10* cells. Two high expression variants H1 (NCT) and H2 (LQI) and WT eGFP constructs are indicated. Letter in superscript indicates amino acid in the second position (A-alanine, V-valine, E-glutamic acid). **d** In vivo analysis of near cognate start codons GUG and UUG eGFP variants. eGFP antibody (JL-8, Clontech), *E. coli* peptide release factor I (αEcRF1), anti-mouse and anti-rabbit HRP-conjugated secondary antibodies were used to visualize the expression of eGFP and normalization of western blot data, respectively. BioRad Precision Plus marker is indicated in images.

We next probed the effects of mRNA and protein stability on the expression of the reporter protein in vitro and in vivo. In particular, we compared the expression of wild type eGFP and AADUAU hexanucleotide variants (M1p1–M1p4) identified by analysis of our reporter library. Western blot analysis (Fig. 4a, Supplementary Fig. 11), the kinetics of in vitro protein synthesis (Supplementary Fig. 12) and endpoint eGFP fluorescence for both in vivo and in vitro experiments (Fig. 4b) confirmed our library results, for which M1p1–M1p4 eGFP variants displayed higher expression levels than WT construct. The expression levels did not correlate with local RNA folding energy as WT eGFP (Δ*G* = −7.70 kcal/mol) construct had less RNA folding energy than M1p1 variant (Δ*G* = −8.50 kcal/mol) but was expressed 6–10 times less in vivo or in vitro (Fig. 4a, b and Supplementary Figs. 9, 12, 13). Similarly, Mp2 (Δ*G* = −8.00 kcal/mol) and Mp3 (Δ*G* = −7.7 kcal/mol) constructs had lower folding energy for mRNA variable region ±30 nucleotides than Mp1 variant (Δ*G* = −8.50 kcal/mol) but were expressed at a lower level both in vivo or in vitro (Fig. 4a, b and Supplementary Figs. 9, 12–13). We also noted that in vitro expression of Mp1–Mp4 constructs showed moderately higher levels (range 3–10 fold higher than WT) when compared to the in vivo expression in *E. coli* BL21 cells (3–6-fold higher than WT), suggesting some contribution of protein degradation and mRNA stability to the observed difference in protein yields. However, in vitro results also indicated that protein and mRNA stability do not correlate to alterations in eGFP expression driven by amino acid identities in position 3–5 and ORF nucleotides 7–15 present in the Mp1–Mp4 constructs. In addition, expression of WT eGFP and two randomly picked reporter constructs coding for NCT and LQI in positions 3–5 maintained the difference in expression ratio regardless of the change in the 2nd amino acid (Fig. 4c) or when a different *E. coli* strain was used for expression (Supplementary Fig. 14). Finally, changing the starting codon (AUG) to near-cognate start codons (GUG, UUG) in three different eGFP variants resulted in the overall reduction of eGFP expression as observed previously^39^, but the relative expression difference between the three sequences was unaffected by the start codon (Fig. 4d and Supplementary Fig. 15). As such, we deduced that expression differences of analyzed eGFP variants were not driven by overall protein or mRNA stability (in vitro experiments) or character of the 2nd amino acid (N-end rule, in vivo experiments)^40^. The difference in the ratio of expression for tested eGFP reporters was maintained despite the usage of different *E. coli* strains or reduced efficiency of start codon recognition during initiation on near-cognate start sites.

### Ubiquitous influence of codons 3–5 on protein synthesis

**Fig. 5:**
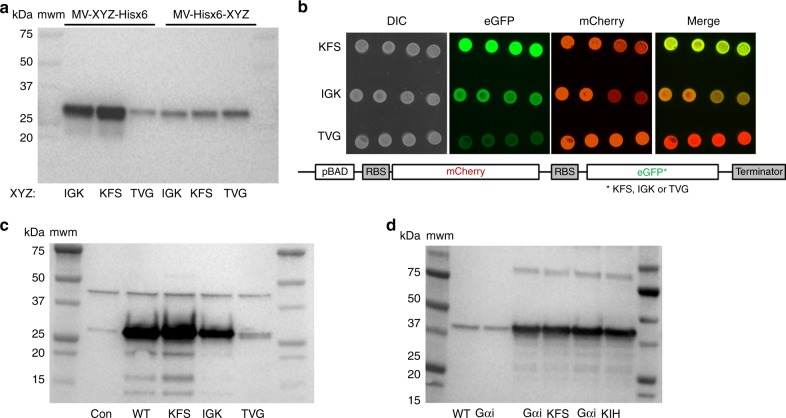
The position and context of the motifs around the initiation codon is critical for protein-synthesis yield. **a** Positional bias in controlling the expression of eGFP constructs with different amino acids in positions 3, 4, and 5. Western-blot analysis of NEB-Pure-Express in vitro expression of eGFP constructs with sequence XYZ (KFS, IGK, and TVG, respectively) as amino acids 3(*X*), 4(*Y*), and 5(*Z*) followed by 6×His tag (MV-*XYZ*-6xHis) or as amino acids 9(*X*), 10(*Y*), and 11(*Z*) preceded by 6×His tag (MV-6×His-*XYZ*). Five percent of in vitro translation reaction is analyzed. **b** Images of *E. coli* colonies expressing mCherry-eGFP polycistronic constructs from pBAD double vector (shown in the schematic). Four colonies of each construct expressing WT mCherry and eGFP constructs with sequence KFS, IGK, and TVG as amino acids inserted in positions 3, 4, and 5 are shown. Dylight 650 (mCherry), Alexa488 (eGFP) and epi white (optical) filters were used to image expression of mCherry and eGFP proteins in *E. coli* colonies. **c**, **d** Simple insertion of different amino acids in recombinant mEOS2 or human Gαi protein constructs can modulate their expression in vivo in *E.coli* BL21 cells, respectively. Wild type (WT) and control samples as well as amino acids in position 3, 4, and 5 for variants of mEOS2 and Gαi proteins are indicated. mEOS2 and Gαi constructs were cloned in pET16b and pBAD vector as C-terminally His-tagged proteins. Proteins were visualized based on their C-terminal 6-Hist tag using Penta-His (Qiagen) antibody. GFP antibody (JL-8, Clontech) is used to visualize the expression of eGFP and BioRad Precision Plus marker is indicated in all images. The same amount of the *E.coli* cells (OD600) was used for Western Blot analysis of in vivo expression of different reporter constructs.

We generated several reporters to test whether our results depend on the position of an amino acid or nucleotide motif with respect to the start codon and are independent of the rest of the reporter sequence. First, we selected three eGFP variants with somewhat different expression levels as determined by analysis of our library and different RNA-folding energies for the variable region ±30 nucleotides (KFS for high, IGK for medium, and TVG for low expression) and inserted a 6× histidine tag between the variable sequence and eGFP sequence (creating constructs MV-XYZ-6xHis-eGFP, where XYZ is KFS, IGK, or TVG). While RNA folding energies changed when the 6× His tag was introduced these constructs still produced the expression profile specified by the GFP score calculated from the library in both in vitro and in vivo experiments (Fig. 5a and Supplementary Fig. 16). However, insertion of the 6× His tag between the second codon and the variable sequence, equalized expression of all constructs both in vivo and in vitro regardless of RNA folding energies (Fig. 5a and Supplementary Fig. 15) arguing again that the position of amino acid and nucleotide motifs at the N-terminus contribute to protein synthesis efficiency. To verify that the MV-KFS-6×His-eGFP protein had the same properties as the WT eGFP protein, we purified both proteins from *E. coli* cells and analyzed their spectral properties (Supplementary Fig. 17). While the addition of three amino acids (KFS) and a 6× His-tag increased overall protein production it did not change either quantum yield (*Q*_SKG_ = 0.72 for WT eGFP, and *Q*_KFS_ = 0.71 for MV-KFS-6×His-eGFP) or absorbance spectra of eGFP variants^41^. As such, the difference in eGFP fluorescence levels between the two transformed *E. coli* cells reflected a difference in protein amounts and not in the folding of the two tested eGFP variants.

To further examine the effects of codons 3–5 on protein expression and impact of the 5′ non-coding sequences we turned towards vectors that express polycistronic mRNAs. The majority of the *E. coli* genes are expressed from polycistronic operons^42^ where expression of each individual transcript is dependent on ORF-centric structures^8^. We tested expression of the same eGFP variants as in previous experiments, however, now cloned as the second ORF in polycistronic operon with mCherry into two different vectors (Fig. 5b and Supplementary Fig. 18). In addition, the two vectors had different promoter, intergenic and ribosome binding (RBS) sequences close to the translation start site. Expression of mCherry reporter was uniform for all constructs regardless of the vector, while eGFP variants were expressed at different levels that correlated with the GFP score determined from our library (Fig. 5b and Supplementary Fig. 18). Noticeably in vivo expression of eGFP variants from both polycistronic vectors, as well as in vitro expression from T7 polymerase transcribed polycistronic mRNAs, recapitulated data using monocistronic reporter (Fig. 3 and Supplementary Figs. 12–18). As such, previously observed differences between eGFP variants were maintained regardless of the polycistronic arrangement, differences in promoter, intergenic (non-coding) sequences or RBS.

Having observed that the addition of 6× His codons downstream of codons 3–5 preserved differences between eGFP variants, we next sought to assess the influence of the rest of the ORF on protein expression. Introduction of the motifs described above in the mEOS2 coding sequence produced the same expression profile as previously determined for KFS, IGK, and TVG motifs (Fig. 5c). Insertion of the high-expressing KFS and KIH motifs in position 3–5 of the N-terminally 10× His tagged human Gα_i_ protein (hGα_i_) resulted in significantly increased expression of recombinant protein (Fig. 5d) even though RNA folding energies were rather similar hGαi KFS (Δ*G* = −4.20 kcal/mol), hGαi KIH (Δ*G* = −6.30 kcal/mol) and hGαi WT (Δ*G* = −5.10 kcal/mol). The two proteins, mEOS2 and hGα_i_, were expressed under different promoters (arabinose vs. T7 promoter, respectively), and had different 5′ untranslated sequences (UTRs) and even the number of nucleotides between ribosome binding sites (RBS) and start codons (12 vs. 7, respectively). Finally, we wondered if the GFP scores could predict the expression level of recombinant human protein that has multiple or alternative start sites. It has been observed that alternative start sites are significantly overrepresented in 5′ regions of genes from multiple species^43^ with the highest frequencies in position 2–9 of the coding sequence^44^. To test the effect of alternative start sites on expression of recombinant protein with alternative start sites, we cloned human RGS2 protein (hRGS2)^45^ in our pBAD vector. We used previously published hRGS2 mutants that had single starting Met codon and Met to Leu codon mutation replacing alternative starting Met codons (Supplementary Fig. 18)^45^. In vitro and in vivo expression of each hRGS2 variant with a C-terminal 6xHis-tag and a single starting Met-codon (M1, M5, M16, and M33) followed the previously established distribution of GFP scores and not local ±30 nucleotides RNA folding energies (Supplementary Fig. 19). Together, these data demonstrate that tested motifs have a strict positional bias (nucleotides 7–15, amino acids 3–5) and are able to modulate protein synthesis efficiency regardless of the differences in the vector (promoter, terminator), upstream non-coding sequence (5′UTR and RBS) or downstream coding sequence (eGFP, mEOS2, hGαi, or hRGS2).

### Early elongation pauses affect protein expression

**Fig. 6:**
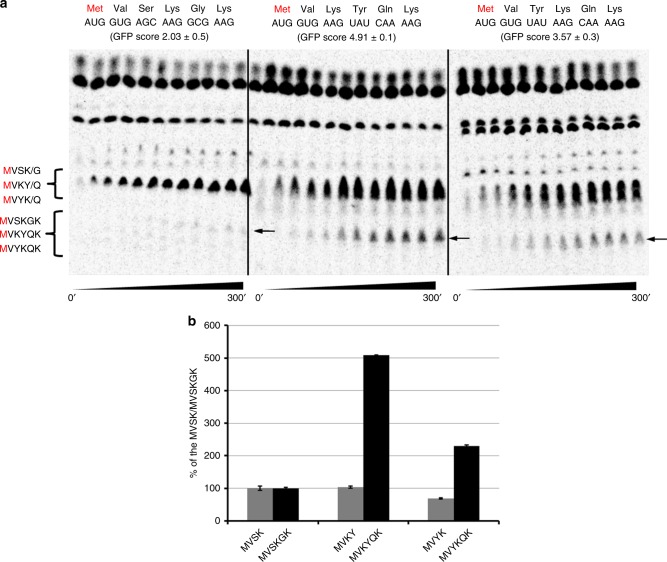
The identity of the first 5 amino acids impacts protein synthesis in a well-defined in vitro translation system. **a** Thin-layer chromatography (TLC) analysis of in vitro peptide synthesis using S35-labeled methionine (M in red). Sequences and GFP scores of tetra-peptides, penta-peptides, and hexa-peptides representing starts of wild type eGFP (MVSKGK) and two high expressing clones MVKYH and MVYKH are indicated. Protein synthesis was initiated from pelleted initiation complexes at time 0 and resolved over time (300 s). Points at 1, 5, 10, 20, 30, 45, 60, 90, 120, 180, 240, 300 s are shown. Migration of tetra-peptide, penta-peptide, and hexa-peptide is indicated. Arrows indicate final hexa-peptide products of the reaction. **b** Analyses of accumulation of the tetra-peptide and hexa-peptide for MVSKGK, MVKYHK, and MVYKHK peptides. Amounts of radioactivity for tetra-peptide and hexa-peptide for the last three time points (180–300 s) were normalized to total radioactivity and plotted in relation to MVSK or MVSKGK peptide amounts. Error bars represent standard deviation.

Our in vitro and in vivo assays as well as our experimental data with different proteins and vectors (Figs. 4, 5 and Supplementary Figs. 12–19) indicate striking differences in the amount of synthesized protein that is driven by nucleotide sequence at position 7–15 and that of the amino acid sequence at residues 3–5 of the open-reading frame. The position of the randomized sequence in our library (Figs. 1–5, Supplementary Figs. 3–19) could affect initiation or early elongation steps of protein expression. To address this possibility, we assayed the efficiency of initiation complex formation and kinetics of peptidyl transfer using a well-defined in vitro *E. coli* translation system^46^. We did not observe any significant difference in the formation of translation initiation complex on 40 nucleotide long messages (13–18% initiation efficiency), resembling either WT eGFP (MVSKG), one of the preferred AAVUAU motifs (MVKYQ) or a permutation of the motif (MVYKQ). However, the yield of protein synthesis from the three initiation complexes varied significantly (Fig. 6a). While the full-length MVSKGK peptide could hardly be observed after 5 min of incubation with ternary complexes and EFG, the MVKYQK peptide was readily detected after only 10 s. Permuted MVYKQK full-length product was also detected albeit with less yield than that seen with the MVKYQK sequence (Fig. 6a). This difference is likely corresponding to the difference in GFP scores between YKQ (GFP score = 3.57 ± 0.28) and KYQ (GFP score = 4.91 ± 0.06) following the observed role of nucleotide and amino-acid composition of codon 3–5 (nucleotides 7–15, amino acids 3–5) in determining the overall efficiency of protein synthesis. Surprisingly, translation of MVSKGK peptide seemed to be aborted or stalled at the incorporation of the 4th or 5th amino acid (Fig. 6a, MVSK and MVSKG products). Quantification of tetra-peptides, translated during the course of last three time points (180–300 s at Fig. 6a), revealed almost identical amounts of synthesized peptides regardless of their amino acid or nucleotide sequences (Fig. 6b, MVSK, MVKY, or MVYK). However, processivity of translating ribosomes beyond tetra-peptides indicated 5-fold and 2.3-fold decrease during the synthesis of MVSKGK peptide relative to that of MVKYQK and MVYKQK, respectively (Fig. 6b). Simple permutation of two codons and changing the order of two amino acids in the starting penta-peptide (MVKYQ vs. MVYKQ), resulted in more than 2-fold reduction of complete MVYKQK peptide compared to MVKYQK peptide (Fig. 6b), again highlighting the amino acid and nucleotide positional bias.

### Codons 3–5 determine the processivity of early elongation

**Fig. 7:**
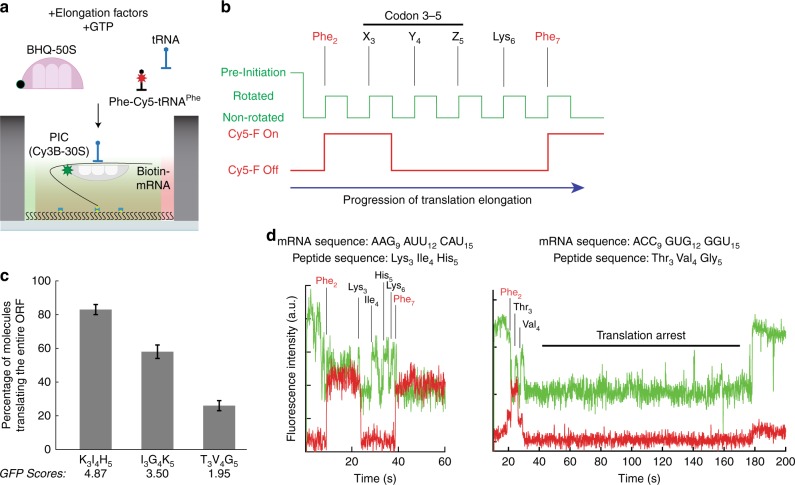
smFRET assay for monitoring translation of codon 3–5. **a** Schematics of zero-mode waveguide (ZMW)-based single-molecule FRET assay to monitor translation. Elongation factors including fluorescently-labeled tRNA (Phe-(Cy5)-tRNA^Phe^) and quencher-labeled large ribosomal subunit (BHQ-50S) are delivered to pre-initiation complex (PIC) with labeled small ribosomal subunit (Cy3B-30S) and mRNA tethered to the ZMWs. **b** Expected fluorescence signal observed from a translating complex utilizing FRET between Cy3B and BHQ-2 on ribosomal subunits, as well as direct excitation of Cy5 on Phe-(Cy5)-tRNA^Phe^. **c** Measured percentage of complete translation events for different codon 3–5 mRNA constructs (*n* = 179 molecules for all; error bars represent s.e. based on the binomial distribution). **d** Representative traces for “processive” translation of K_3_I_4_H_5_ (Left) and “abortive” translation of T_3_V_4_G_5_ (Right).

To investigate how the observed changes in overall protein expression are related to translating codons 3–5, we used a single-molecule Förster resonance energy transfer (smFRET)-based assay to monitor translation of multiple (6–12) codons in real-time (Fig. 7a, b; Supplementary Fig. 20)^47,48^. During translation, successful tRNA decoding and translocation steps induce coupled ribosome conformational changes to and from non-rotated and rotated states. By site-specifically labeling ribosomal subunits with a pair of FRET probes, transitions between non-rotated and rotated states of each translating ribosomes are monitored as FRET efficiency changes between attached Cy3B and BHQ-2. In addition to the FRET signal reporting the ribosome conformation, the progression of translation was independently followed by the binding and departure of fluorescently labeled Phe-specific tRNA to present Phe codons within mRNA coding sequences. To tolerate high concentration of fluorescently labeled tRNA in the assay, we utilized a zero-mode waveguide-based (ZMW) experimental platform^49^, which limits the background signal. Based on the rotated and non-rotated state transitions and state lifetimes observed for >100 translating ribosomes, the rate and processivity for translating each codon were calculated.

Using this setup, we tested three different mRNA sequences in positions 3–5 based on their GFP scores: K_3_I_4_H_5_ was used for high, I_3_G_4_K_5_ for medium and T_3_V_4_G_5_ for low expression in otherwise identical sequence (Fig. 7b). Comparing translation of the high (K_3_I_4_H_5_) and low (T_3_V_4_G_5_) expression mRNA constructs, we have observed a substantial alteration in translation elongation processivity (Fig. 7c; Supplementary Fig. 21), defined as the percentage of ribosomes that translated the entire ORF after the first elongation on the second codon (Phe_2_ or F_2_). The majority of ribosomes (84%) translated the entire ORF in K_3_I_4_H_5_ construct (Fig. 7c, d). However, only 27% of ribosomes reached the in-frame stop codon while translating the T_3_V_4_G_5_ construct (Fig. 7c). The majority of ribosomes stopped translation after the incorporation of 3rd (T) and 4th (V) amino acid (Fig. 7d). The experiment with the I_3_G_4_K_5_ construct revealed intermediate ribosome processivity (54% of ribosomes translating the entire ORF), showing the ribosomes arresting after incorporating amino acids 3 (I) and 4 (G) similar to the T_3_V_4_G_5_ construct. However, for ribosomes that passed this “processivity barrier” at amino acids 3 and 4, translation elongation rates (measured as both non-rotated and rotated state lifetimes for coupled tRNA decoding and translocation steps) for codon 3–7 were comparable across different mRNA constructs (Supplementary Fig. 21), indicating possible existence of irreversible branch-points to abortive translation during the first five codons, rather than a gradual slow-down of elongation over codons 3–5. The low processivity of translation observed for T_3_V_4_G_5_ construct was readily replicated in additional experiments performed at a different temperature as well as at a different translational factor concentration (Supplementary Fig. 22). The positional bias of KIH vs. TVG were further tested by moving from codons 3–5 to 9–11 after adding six histidine for codons 3–8 (Supplementary Fig 23), which decreased the differences in processivity from 84 and 27% at codons 3–5 to 58 and 35% at codons 9–11 for KIH and TVG constructs, respectively. Taken together, these data suggest that the ribosome arrest at codons 3–5 is responsible for the translation efficiency differences observed in our study, where the processivity differences measured for a single round of translation are likely to be amplified in multiple rounds of translation in vivo.

**Fig. 8:**
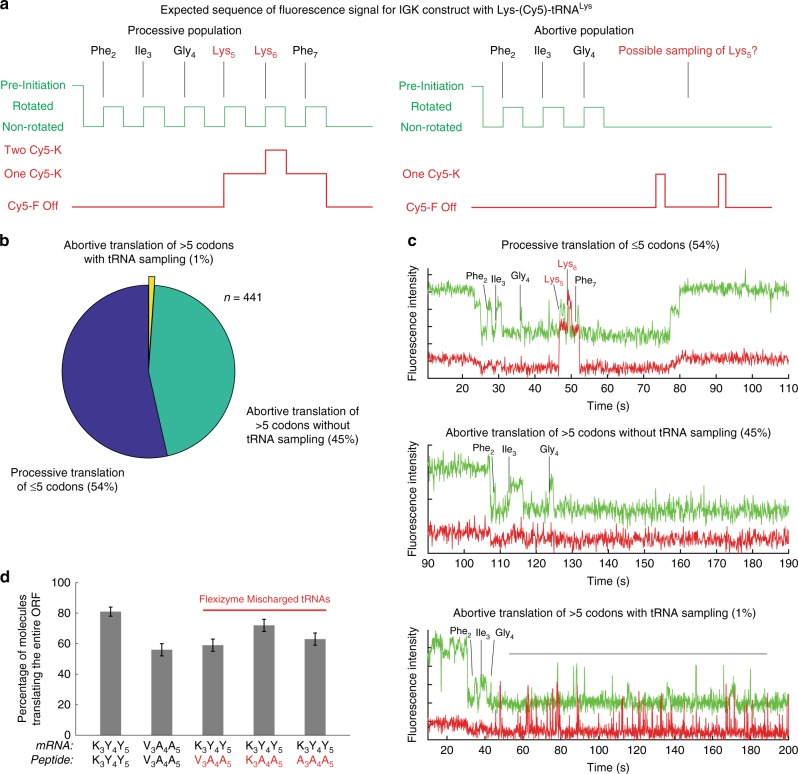
Parsing tRNA and amino acid contributions to abortive translation of codon 3–5. **a** Expected fluorescence traces for successful and abortive translation. A possible sampling of tRNA to the A site may occur on the arrested ribosomal population, resulting in multiple binding event of Cy5-labeled specific tRNA such as Lys-(Cy5)-tRNA^Lys^. **b** A pie-chart of different populations observed in the experiment. **c** Representative traces for each population. **d** Measured percentage of complete translation events for K_3_Y_4_Y_5_ and V_3_A_4_A_5_ mRNA codons with a correct peptide, and for translating K_3_Y_4_Y_5_ mRNA codons with V_3_A_4_A_5_, K_3_A_4_A_5_ or A_3_A_4_A_5_ peptide sequences using Flexizyme-mischarged tRNAs (*n* = 161, 147, 156, 131, and 142 molecules from left to right; error bars represent s.e. based on the binomial distribution).

To probe the ribosomal structural state during the abortive translation at early codons, we monitored stable binding of the incoming aa-tRNA to the A site. As in previous experiments, we have used the ribosome conformational signal to detect the conformation of translating ribosomes, but used Cy5-labeled Lys-tRNA (Lys-(Cy5)-tRNA^Lys^) to probe A-site tRNA binding while translating the I_3_G_4_K_5_ nascent-peptide sequence (Fig. 8a–c). Analysis of 441 molecules indicated three classes of translation events (Fig. 8a–c): Complete translation of ORF (54%), aborted translation after 4th amino acid (G_4_) without Lys-(Cy5)-tRNA^Lys^ sampling (defined as tRNA binding longer than >100 ms^50^ the A-site Lys codon) (45%), and one that exhibited Lys-(Cy5)-tRNA^Lys^ sampling in aborted translation (1%). Considering that a majority of arrested ribosomes exhibited a non-rotated-like conformational state without (>100 ms lifetime) A-site tRNA sampling necessary for a processive elongation, we hypothesize that the ribosome is in a non-canonical structural state that cannot make a stable interaction among rRNA monitoring bases and codon-anticodon duplex necessary for further elongation^50^. Such state may be a result of different pathing of an mRNA as well as a nascent-peptide molecule within the ribosome, possibly similar to the previously observed interaction among the ErmCL nascent-peptide, the ribosome exit tunnel and the antibiotic erythromycin^51^, allosterically affecting the decoding site of the ribosome.

### Early elongation is influenced by the nascent peptide

To understand a relative contribution of a peptide sequence at codons 3–5 compared to their respective nucleotide sequence in determining the overall translation efficiency, we used specifically mischarged tRNAs to modify the nascent peptide sequence without altering the mRNA sequence. Among different tripeptide sequences on codons 3–5, we chose K_3_Y_4_Y_5_ and V_3_A_4_A_5_ due to their respective high and medium GFP scores, as well as the availability of mischarged-tRNA reagents. When tested on the previously described single-molecule assay, translation of K_3_Y_4_Y_5_ was highly processive (81% of ribosomes translated the entire ORF; Fig. 8d and Supplementary Fig. 21), whereas translation of V_3_A_4_A_5_ exhibited an intermediate ribosome processivity (56%, Fig. 8d). Next, purified tRNA^Lys^ and tRNA^Tyr^ were respectively mischarged with valine or alanine amino-acids using flexizyme reaction^52^, and used to translate K_3_Y_4_Y_5_ mRNA construct. Surprisingly, changing only the nascent-peptide sequence to V_3_A_4_A_5_ altered the processivity of translating K_3_Y_4_Y_5_ codons to 59% (Fig. 8d), similar to that of translating V_3_A_4_A_5_ codons. Using the same K_3_Y_4_Y_5_ mRNA construct, processivity for two more nascent-peptide sequences (K_3_A_4_A_5_ and A_3_A_4_A_5_) were measured (72 and 63%, respectively) (Fig. 8d), where the presence of K amino-acid at position 3 may have contributed in increasing the processivity following the K|N-Y|I peptide sequence motif. Our result shows that the codons 3–5 amino-acid identities in conjunction with the mRNA sequences on positions 7–15 contribute to the processivity of translation elongation on early codons, which in turn may determine the overall translation efficiency and protein expression.

## Discussion

In summary, we show that the efficiency of protein synthesis is strongly dependent on the nucleotide sequence positions 7–15 and the resulting amino acid positions 3–5 in the nascent peptide, in addition to the overall mRNA structure and codon content. The expression levels of 213,708 eGFP variants with randomized nucleotide and amino acid sequences in those positions resulted in substantial differences in fluorescence and protein levels. The effect of the assayed sequences was dependent on both nucleotide and amino acid sequence, which suggests that a combination of tRNA, mRNA, ribosome, and nascent polypeptide chain interactions define the efficiency of protein synthesis at the very N-terminus. We found that the efficiency of protein synthesis can be enhanced by changing the codons 3–5 for several recombinant proteins, regardless of their downstream mRNA and protein sequence, the expression vector used, and in vitro or in vivo expression conditions.

**Fig. 9:**
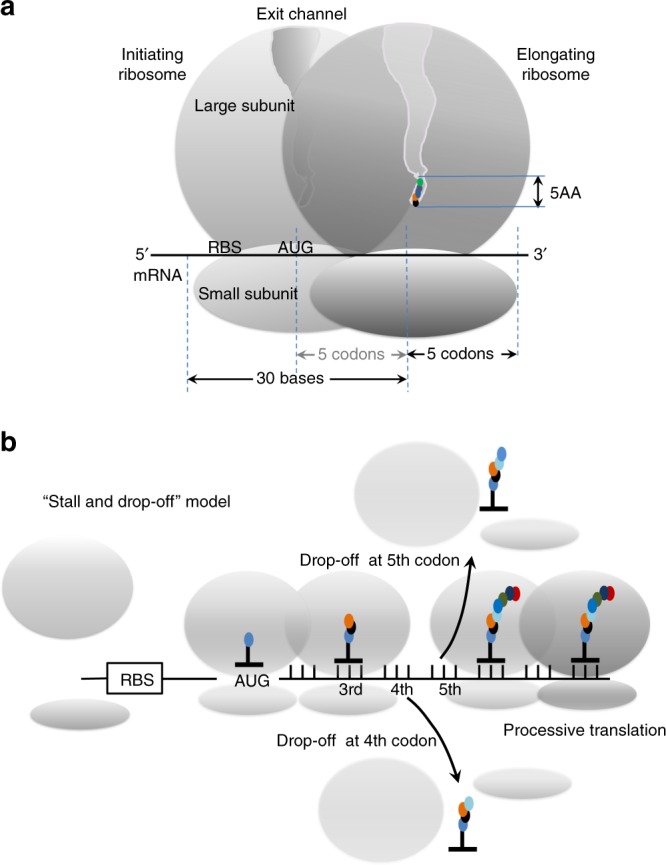
Model for translational regulation by the identity of N-terminal sequence. **a** Schematic representing the initiating and elongating ribosome footprints as well as a movement during the translation of the first five codons described in this manuscript. **b** “Stall and drop-off” model in translational regulation by the identity of N-terminal sequence. Arrows indicate stalled and abortive translation on non-processive peptides followed by ribosome recycling and peptidyl-tRNA drop-off at codons 4 and 5. Start codon (AUG), ribosome binding site (RBS), ribosomal subunits, peptide exit channel, ribosome footprint, peptidyl-tRNAs, amino acids as well as position of detrimental 3–5 codons is indicated.

Our in vitro assays revealed that the varied protein expressions were not due to changes in the initiation, but instead related to elongation of the 4th and 5th codon. The probability of the translation arrest while synthesizing N-terminal penta-peptide may govern the overall efficiency of protein synthesis. This is in agreement with multiple previous studies that argue for the importance of N-terminal sequences in determining the efficiency of protein synthesis^11,15,19,20,29^. Translation arrests at early codons were similarly observed in certain minigenes^53^, *E.coli* peptides in the presence^54^ or absence^55,56^ of macrolide antibiotics, and ribosome profiling studies in yeast and mammalian cells^26,30^. We observed that the majority of arrested ribosomes exhibit a non-rotated-like conformational state without A-site-tRNA sampling (Fig. 8a–c). This non-canonical state is probably induced by the nascent-peptide interacting with the ribosome exit channel to alter the conformation of the peptidyl transfer center, precluding accommodation of the next A-site tRNA. This arrest may resolve through peptidyl-tRNA drop-off and subsequent ribosome recycling (Fig. 9). The conservation of the ribosome peptidyl-transfer center^57^ suggests that a similar mechanism operates in other organisms as well, including Eukaryotes. Indeed, we observed enrichment of codons 3–5 with high GFP-scores in natural transcripts from both *E.coli* and yeast (Supplementary Fig. 24).

The early elongation gate was important in synthesizing proteins at alternative start sites. Our *E.coli* and in vitro data on variation of the protein expression levels from alternative start sites in the recombinant human RGS2 protein is almost identical to data obtained from human cells^45^. One could envision that the processivity of early elongation may contribute to the amounts of protein isoforms initiated at alternative start sites within a single transcript. The differences in efficiency of protein synthesis through N-terminal penta-peptides among alternative start sites would add to the overall complexity of alternative start site selection in eukaryotic cells^43,44,58^. Another example is differential expression of translationally coupled ORFs in certain multiprotein complexes or proteins from biosynthetic pathways that are often encoded in polycistronic operons^23^. Variation in expression of multiple proteins from polycistronic transcript could be achieved by differing processivity of N-terminal penta-peptides associated with each individual ORF. The ratio of individual members of heterogeneous multimeric complexes could be tuned through the same mechanism. It is unlikely that whole transcriptomes underwent such an optimization; rather, it is most probable that independent mutations removed translational blockages in highly expressed genes. For example, EF-Tu protein which is a product of *tufA* gene in *E.coli* and most abundant *E.coli* protein. It starts with MSKEK penta-peptide which is close to the top of our GFP scores (GFP score 4.44 ± 0.37). The other example is *rpiB* gene (coding for ribose-5-phosphate isomerase B) which was identified as highly expressed *E.coli* gene through absolute protein expression measurements (APEX) study. RpiB protein has unusual high protein to mRNA ratio in E.coli cells. The starting penta-peptide of RpiB protein is MKKIA (GFP score 4.71 ± 0.07) which resembles one of identified K|N-Y|I motifs (nucleotide sequence AAVAUU).

Our findings elucidate at least in part the importance of previously observed translational ramps^5–15^, which have been proposed to facilitate efficient mRNA translation by staging elongating ribosomes in such a way as to prevent detrimental collisions during later rounds of elongation. Our findings suggest that codons 3–5 may serve as a gate that controls mRNA translation efficiency differently than originally proposed as a translational ramp encompassing the first 15–30 codons^5,6,9^ (Fig. 9). The reduced processivity of ribosome on non-favorable codons and amino acids in position 3–5 would decrease ribosome collisions along the coding sequence, and act as a gate to potentially reduce the detrimental activation of ribosome quality control mechanisms dependent on collided ribosmes^29^. However, ribosome pausing on codons 3–5 and potential peptidyl-tRNA drop-off lowers overall translational efficiency and translation initiation rates of such transcripts. On the other hand, highly processive codons and amino acids in positions 3–5 would increase the possibility of ribosome collisions along during later rounds of elongation. In such a scenario, positive effects of the short elongation ramp could be counteracted by negative effects of ribosome collisions that would lead to activation of ribosome quality control mechanisms^29^. In these cases, translation initiation rates would play a more detrimental role in staging elongating ribosomes along the transcript. The motifs that we describe here will help in removing some translational blockages, assist in creating tools for higher expression of recombinant and industrial proteins as well as for further studies on how ribosomal early elongation dynamics influence protein synthesis.

## Methods

### Construction of library

To create the EGFP library we used optimized EGFP sequence (codon optimization tool from IDT DNA and http://www.kazusa.or.jp/codon/):

atggtcagcaagggcgaggagctgttcaccggggtggtgcccatcctggtcgagctggacggcgacgtcaacggccacaagttcagcgtgtccggcgagggcgagggcgatgccacctacggcaagctcaccctcaagttcatctgcaccaccggcaagctgcccgtgccctggcccaccctcgtgaccaccctgacctacggcgtgcagtgcttcagccgctaccccgaccacatgaagcagcacgacttcttcaagtccgccatgcccgaaggctacgtccaggagcgcaccatcttcttcaaggacgacggcaactacaagacccgcgccgaggtgaagttcgagggcgacaccctggtgaaccgcatcgagctgaagggcatcgacttcaaggaggacggcaacatcctggggcacaagctggagtacaactacaacagccacaacgtctatatcatggccgacaagcagaagaacggcatcaaggtgaacttcaagatccgccacaacatcgaggacggcagcgtgcagctcgccgaccactaccagcagaacacccccatcggcgacggccccgtgctgctgcccgacaaccactacctgagcacccagtccgccctgagcaaagaccccaacgagaagcgcgatcacatggtcctgctggagttcgtgaccgccgccgggatcactctcggcatggacgagctgtacaagtaa.

The sequence of library was amplified using primers EGFP-lib For and EGFP-Rev using Phusion—HF (NEB).The PCR product was purified using Nucleospin Gel and PCR cleanup kit (Macherey Nagel) prior to digestion with (NcoI—For) and (XhoI-Rev). The digested PCR product was ligated into digested pBAD low copy vector. The ligation product was purified using Nucleospin Gel and PCR cleanup kit (Macharey Nagel) and desalted using Illustra Microspin G-25 Columns (Thermo Fisher). The purified and desalted ligation product was then electroporated into high efficiency 5-alpha *E.coli* cells (NEB). The cells were grown overnight on LB-Carbenicillin plates and then, ~2 × 10^6^ colonies were scraped from the plates and collected in LB-media. An equal volume of 50% glycerol was added to the liquid culture and the cells were frozen at −80 °C.

### Cell sorting

For each FACS experiment, one vial (5 ml) of cryopreserved cells was thawed and grown in LB media with carbenicillin for 90 min. The cells were centrifuged (3000 × *g* for 5 min), media was removed and cells were then induced with the addition of fresh media supplemented with 0.2%% l-arabinose for 3 h. After induction, the culture was pelleted by centrifugation at 3300 × *g* for 10 min and washed with PBS, followed by second centrifugation and a final resuspension in PBS. The cells were sorted by level of GFP expression into five bins using Aria III flow cytometer (BD Biosciences) with median GFP fluorescence of 20, 120, 600, 3600, and 12,000. LB was added to the sorted cells and they were grown at 37 °C for 2 h prior to plasmid isolation using PureLink HiPure Miniprep Kit (Thermo Fisher).

### Illumina library preparation

PCR was performed with primers Lib_Amp_F and Lib_Amp_R and an equal mass of the plasmid isolated from each sorted bin using Phusion-HF MM (98 °C for 1 min, 22 cycles: 98 °C for 10 s, 55 °C for 30 s, 72 °C for 30 s, and 72 °C for 5 min) in separate reactions. The amplicon was purified using Nucleospin Gel and PCR cleanup kit (Macherey Nagel) and then digested with NcoI and XhoI. The digested product was purified as done previously and ligated into Illumina adapters. It was then amplified using Il_Enrich_F and Il_Enrich_R using Phusion HF MM (98 °C for 1 min, 21 cycles: 98 °C for 10 s, 66 °C for 30 s, 72 °C for 30 s, and 72 °C for 5 min). The product was subsequently resolved by agarose gel electrophoresis, and the appropriate sized band was excised and purified. The Illumina library was multiplexed and run on four lanes of the Illumina NextSeq System.

### Sequencing analysis

Counts for each triplet codon sequence within each FACS sorted bin, and the input plasmid pool were determined from our sequencing data sets. Sequences with less than ten total counts across all bins were removed. We normalized the number of counts for each construct within a bin by the total number of counts in the respective bin. We determined the “GFP Score” by obtaining a weighted average of counts across all of the bins for a given sequence. In short, the ratio of the normalized counts within each bin and the total across all five bins for a given sequence was multiplied by the bin number which corresponds to increased GFP expression. The average of these weighted values for each sequence was then determined to give a “GFP Score”:

GFP score = (Normalized_Counts_bin_1/total_readscounts*1) + (Normalized_Counts_bin_2/total_counts*2) + (Normalized_Counts_bin_3/total_counts*3) + (Normalized_Counts_bin_4/total_counts*4) + (Normalized_Counts_bin_5/total_counts*5).

As such, one represents minimal and five maximal eGFP score; wild type eGFP sequence has a score of 2.57 ± 0.00. We compared “GFP Score” to various mRNA (GC or AT content) and peptide sequence attributes (charge and hydrophobicity) in R using custom scripts or previously described packages (*peptides*), respectively.

For comparison of tAI with “GFP Score” we determined the tAI of all possible triplet codon sequences using *CodonR* (https://github.com/dbgoodman/ecre_cds_analysis/tree/master/codonR). To identify mRNA sequence motifs we used the R package *motifRG*. Sequences with a “GFP Score” above four were considered “high” and sequences with a “GFP Score” below three were considered “low”. The same stratification was used for identifying peptides associated with mRNA sequences with high “GFP Score” using the R package peplib. All scripts used for analysis are available at the Github repository under MIT license (https://github.com/cottrellka/EGFP_library_seq). Sequencing data is available at the Sequence Read Archive under: https://www.ncbi.nlm.nih.gov/Traces/study/?acc=PRJNA590742.

Folding energies were calculated with RNA Fold from Vienna RNA package for all combinations of three-codon sequences (262,144 in total) using −30 nucleotides as the left flank and +30 nucleotides as the right flank^38^.

### **In vivo** constructs expressions

Modified and wild type mEOS, eGFP and human G protein subunit alpha i1 (Giα; NM_002069) construct DNA were created by PCR using forward primers that code for the certain sequence extracted from our EGFP library expression found in the FACS experiment (KFS, KYY, KIH—high expression, IGK—moderate expression, TVG—low expression). Regulator of G protein signaling 2 (RGS2; NM_002923) constructs were amplified by PCR reaction from previously described constructs^59^. PCR products were cloned in the pBAD, petDUET or pET16b vector, transformed into top ten *E. coli* cells and sequenced for the correct clones. pBAD double vector for polycistronic operon was created using original pBAD vector by introduction of the ribosome binding site (RBS), second ORF and multiple cloning site 16 nucleotides below the stop codon of the previous ORF. Correct plasmids were transformed to *E. coli* cells for in vivo expression (TOP10, BL21 DE3, DH5α, W3110, XAC *E. coli* cells were used for expression experiments). Three colonies were picked off the plates and grown overnight. Their optical density was measured and equalized to 0.1 OD at 600 nm, once they reached OD_600_ of 0.5 colonies were induced with the addition of l-arabinose to the final 0.2% in LB-media. The expression of fluorescent proteins was followed by eGFP fluorescence and normalized to the number of cells for monocistronic reporters or normalized to the fluorescent of mCherry in case of polycistronic operons. After 3 h of induction, the same number of cells (based on OD_600_) was centrifuged and re-suspended in 2× SDS buffer. Samples were heated at 95 °C for 5 min, after which they were frozen at −20 °C for further use. The same volume of samples were loaded on 4–16% gradient SDS-PAGE gels and analyzed by western blot analysis using EGFP (JL-8; Clontech),penta-HIS (QIAGEN) or α-RF1 E.coli (Zaher Lab) antibodies. Anti-mouse or anti-rabbit HRP conjugated antibodies were used as secondary antibodies.

### **In vitro** constructs expressions

PCR products from pBAD or pet16 cloned constructs were used as templates for NEB PURE or PUREFREX 2.*0* in vitro translation reactions. In short, DNA constructs were amplified using Phusion—HF (NEB) kit using T7 forward primer and gene-specific reverse primer. The PCR product were analyzed on agarose gels and purified using the Zymo Clean DNA gel extraction kit. Equal amounts of DNA (50–150 ng) were used in in vitro reactions. If noted PCR products were used to synthesize RNA using T7 polymerase kit (NEB), purified using NEB RNA purification kit and equal amount of purified RNA was used for in vitro reaction (1–3 µg). In vitro protein synthesis was conducted for 2.5 h at 37 °C if not noted differently. In the case of fluorescent proteins translation was followed in parallel by fluorescence reading using for 2.5 h in 1 min intervals. Same amount of samples were loaded on SDS PAGE gels and western blot analyses were performed as described for in vivo expression experiments.

### Spectroscopy experiments

A Thermo Scientific™ Pierce™ BCA™ Protein Assay (code 10678484) has been used to have an estimate of the total protein concentration compared to a protein standard. All spectroscopic experiments have been carried out with an UV–VIS Fluorescence Spectrophotometer ISS K2. The absorbance spectrum was measured between 350 and 550 nm. Relative quantum yield is generally obtained by comparing the intensity of an unknown sample to that of a standard. The quantum yield of the unknown sample can be calculated using: Q = Q_R I/I_R 〖OD〗_R/OD *n*^2/(*n*_R^2), where Q is the quantum yield, I is the integrated intensity, n is the refractive index, and OD is the optical density. “R” refers to the reference fluorophore of known quantum yield (in this case fluorescein). Since the end-point method is not accurate for the calculation of the quantum yield, we prepared solutions within the range of 0–0.01 ODs, by subsequent dilutions of the different proteins to calculate the quantum yield using the gradients determined for the sample and the reference. In this case, quantum yield is given by: Q = Q_R (Grad/〖Grad〗_R)(*n*^2/(*n*_R^2)) where Grad is the gradient obtained from the plot of the integrated fluorescence intensity vs. optical density (see Supplementary Fig. 17). Absorbance and concentration of the eGFP variants was calculated for the molecular weight of approximately 27 kDa.

### Formation of ribosomal initiation complexes

To generate initiation complexes (IC), the following components were incubated at 37 °C for 30 min: 70S ribosomes (2 µM), IF1, IF2, IF3, f-[35 S]-Met-tRNAfmet (3 µM each), mRNA (6 µM) in polymix buffer containing GTP (2 mM). The complexes were then purified away from free tRNAs and initiation factors over a 500 µL sucrose cushion composed of 1.1 M sucrose, 20 mM Tris-HCl pH 7.5, 500 mM NH4Cl, 0.5 mM EDTA, and 10 mM MgCl_2_. The mixture was spun at 287,000 × *g* at 4 °C for 2 h, and the resulting pellet was resuspended in 1× polymix buffer and stored at −80 °C. To determine the concentration of IC, the fractional radioactivity that pelleted was recorded.

### Kinetics of peptidyl transfer

Ternary complexes were formed as described previously^60^. Briefly, EF-Tu (10 µM final) was incubated with GTP (10 mM final) and a mix of aminoacyl-tRNAs (including valine, serine, lysine, alanine, glutamine, arginine, glutamic acid, methionine, and tyrosine) in polymix buffer for 15 min at 37 °C. The ternary complex mixture was then combined with an equivalent volume of IC at 37 °C. The reaction was stopped at different time points using KOH to a final concentration of 500 mM. Peptide products were separated from free fMet using cellulose TLC plates that were electrophoresed in pyridine-acetate at pH 2.8^46^. The TLC plates were exposed to a phosphor-screen overnight, and the screens were imaged using a Personal Molecular Imager (PMI) system.

### ZMW-based single-molecule fluorescence assay

Overall experimental setup (using Pacific Bioscience RSII) and biological reagents have been prepared as described previously^47–49^. Briefly, each small and large subunit were mutated to include a weakly forming RNA hairpin at helix 44 and helix 101, which was used to attach Cy3B/BHQ-2 labeled DNA oligonucleotides via RNA/DNA hybridization (labeled DNA oligonucleotides purchased from TriLink Technologies). Individual tRNA species used were purchased from Chemical Block Ltd. tRNA^Lys^ or purified from bulk *E. coli* tRNA^61^. tRNA^Phe^ was labeled at acp^3^U47 position with Cy5 using NHS chemistry as previously described^62^, with Cy5-NHS-ester purchased from GE Healthcare. Synthesis and purification of activated Ala-and Val-DBE (3,5-dinitrobenzyl esters) derivatives was done using detailed protocol^63^. Aminoacylation of Lys- and Tyr-tRNA (Sigma Aldrich) with synthesize Val-and Ala-DBE derivatives was done using dFx ribozyme (IDT RNA oligoes) as described by Zhang and Ferre-D’Amare, 2014^64^. 5′-Biotinylated mRNAs used for single-molecule translation assay are purchased from Horizon Dharmacon. Translational factors, ribosomal S1 protein, and aminoacylated tRNAs were prepared as previously reported. All single-molecule experiments were conducted in a Tris-based polymix buffer consisting of 50 mM Tris-acetate (pH 7.5), 100 mM potassium chloride, 5 mM ammonium acetate, 0.5 mM calcium acetate, 5 mM magnesium acetate, 0.5 mM EDTA, 5 mM putrescine-HCl, and 1 mM spermidine, with additional 4 mM GTP.

Immediately before each single-molecule experiment, small and large ribosomal subunits were mixed with respective fluorescently labeled DNA oligonucleotide at 1:1.2 stoichiometric ratio in the previously described polymix buffer. Small ribosomal subunits were subsequently mixed with S1 ribosomal protein at 1:1 stoichiometric ratio, and subsequently mixed with biotinylated-mRNA, initiation factor 2, amino-acylated formyl-methionine tRNA at 1:2:13:4 in the presence of 4 mM GTP to form 30S Pre-Initiation Complex (30S PIC). 30S PIC was diluted to 10 nM in the polymix buffer supplemented with 4 mM GTP and the imaging mix (2.5 mM of PCA (protocatechuic acid), 2.5 mM of TSY, and 2× PCD (protocatechuate-3,4-dioxygenase), purchased from Pacific Bioscience; PCD added last), and incubated in the zero-mode waveguide chip treated with Neutravidin at room temperature. After immobilizing the pre-initiation complex, the chip was washed three-times using the same buffer without the complex to remove unbound complexes, and loaded onto the RSII instrument. At the same time, the delivery solution, a polymix buffer supplemented with 4 mM GTP, the imaging mix, varying concentration of tRNA ternary complexes (labeled or unlabeled), varying concentration of EF-G, and 200 nM of the BHQ-2 labeled large ribosomal subunit was prepared, and loaded onto the instrument. In general, final concentration of purified 50 nM of Phe-(Cy5)-tRNA^Phe^ (50 nM of Flexizyme-charged tRNA for applicable experiments), 0.7 µM of total delta-Phe aa-tRNA (total tRNA charged with all amino-acids except Phe; tRNA from Roche) and 100 nM of EF-G were used. A higher concentration of factors or different set of tRNAs were used as indicated in each experiment.

At the start of the experiment, the instrument delivered the delivery solution to the chip, and recorded an 8-min movie with frame rate ten frame per second, illuminated by 60 mW per mm^2^ of 532-nm laser and 10 mW per mm^2^ of 642-nm laser. Experiments were performed with the chip temperature clamped to the specified temperature, usually ranging from 20 to 30 °C. Resulting movies were analyzed using in-house-written MATLAB (MathWorks) scripts, as previously described. Briefly, traces from each zero-mode waveguide wells were manually filtered based on the presence of both fluorophores at different time points (signal from immobile fluorophores on the ribosome was expected to be present at the beginning of the movie, while signal from fluorophores attached to tRNA was expected not to be) and a single photobleaching step for each fluorophores. Filtered traces were manually assigned to rotated state and non-rotated state after the subunit joining event, cross-correlated with the labeled tRNA binding signals. From assigned traces, both rotated and non-rotated state lifetimes were calculated by fitting a single-exponential distribution to the measured state lifetimes using maximum-likelihood estimation in MATLAB.

### Reporting summary

Further information on research design is available in the Nature Research Reporting Summary linked to this article.

## Supplementary information

Supplementary Information

Reporting Summary

Description of Additional Supplementary Files

Supplementary Data 1

Supplementary Data 2

Supplementary Data 3

Supplementary Data 4

Supplementary Data 5

## Data Availability

The data that support this study are available from the corresponding author upon reasonable request. Source data for Figures are included in a Source Data file and as Supplementary Figs. 25–34. Sequencing data is available at the Sequence Read Archive under code PRJNA590742.

## Source data

Source Data
