# Supplementary material for: A short translational ramp determines the efficiency of protein synthesis
Source: Nat Commun. 2019 Dec 18;10:5774. doi: 10.1038/s41467-019-13810-1 (PMC6920384; doi:10.1038/s41467-019-13810-1)
Supplement: Supplementary file 1 — Supplementary Information [file 41467_2019_13810_MOESM1_ESM.pdf]

Supplementary Material

Short translational ramp determines efficiency of protein synthesis

Manasvi Verma[1,*], Junhong Choi[2,3,6*], Kyle A. Cottrell[1,*], Zeno Lavagnino[1,7], Erica N. Thomas[4], Slavica Pavlovic-Djuranovic[1], Pawel Szczesny[5], David W. Piston[1], Hani Zaher[4], Joseph D. Puglisi[2], Sergej Djuranovic[1,#]

* equally contributed

# correspondence: sergej.djuranovic@wustl.edu

Affiliations:

[1] Department of Cell Biology and Physiology, Washington University School of Medicine, 600 South Euclid Avenue, Campus Box 8228, St. Louis, MO 63110, USA

[2] Department of Structural Biology, Stanford University School of Medicine, Stanford, California 94305– 5126, USA;

[3] Department of Applied Physics, Stanford University, Stanford, California 94305– 5126, USA;

[4] Department of Biology, Washington University, St Louis, Missouri 63105, USA

[5] Institute of Biochemistry and Biophysics Polish Academy of Sciences, Department of Bioinformatics, Warsaw, Poland

[6] current address: Department of Genome Sciences, University of Washington, Seattle, WA, USA

[7]current address: Experimental Imaging Center, IRCCS Ospedale San Raffaele, Milan, Italy

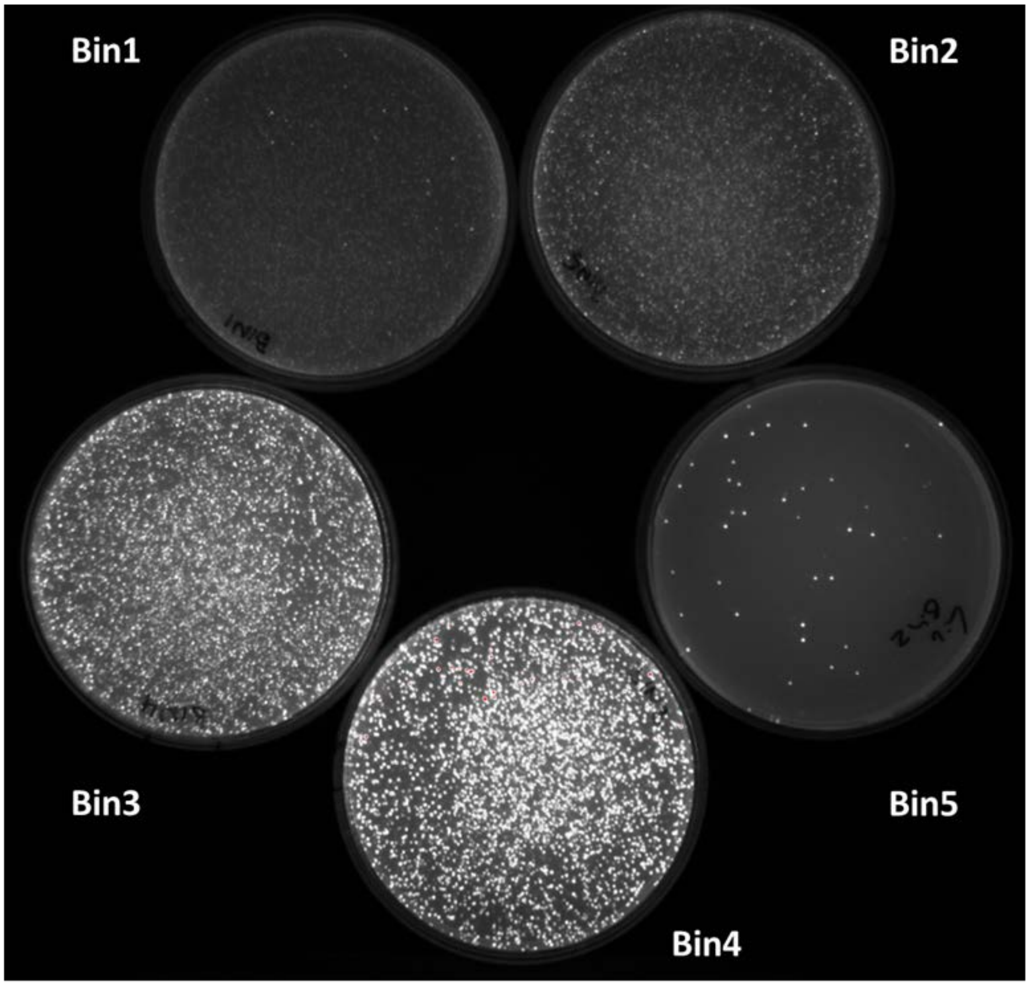

Supplementary Figure 1 | FACS Sorted DH5α expressing the GFP library. Image of arabinose induced *E. coli* colonies separated using fluorescence-activated cell sorting (FACS) into 5 bins. Each bin represents approximately 24% of the whole cell population depending on eGFP expression with exception of bin 5. Bin 5 represents 2.5% of the *E.coli* cells with highest eGFP expression based on relative fluorescence values (RFUs).

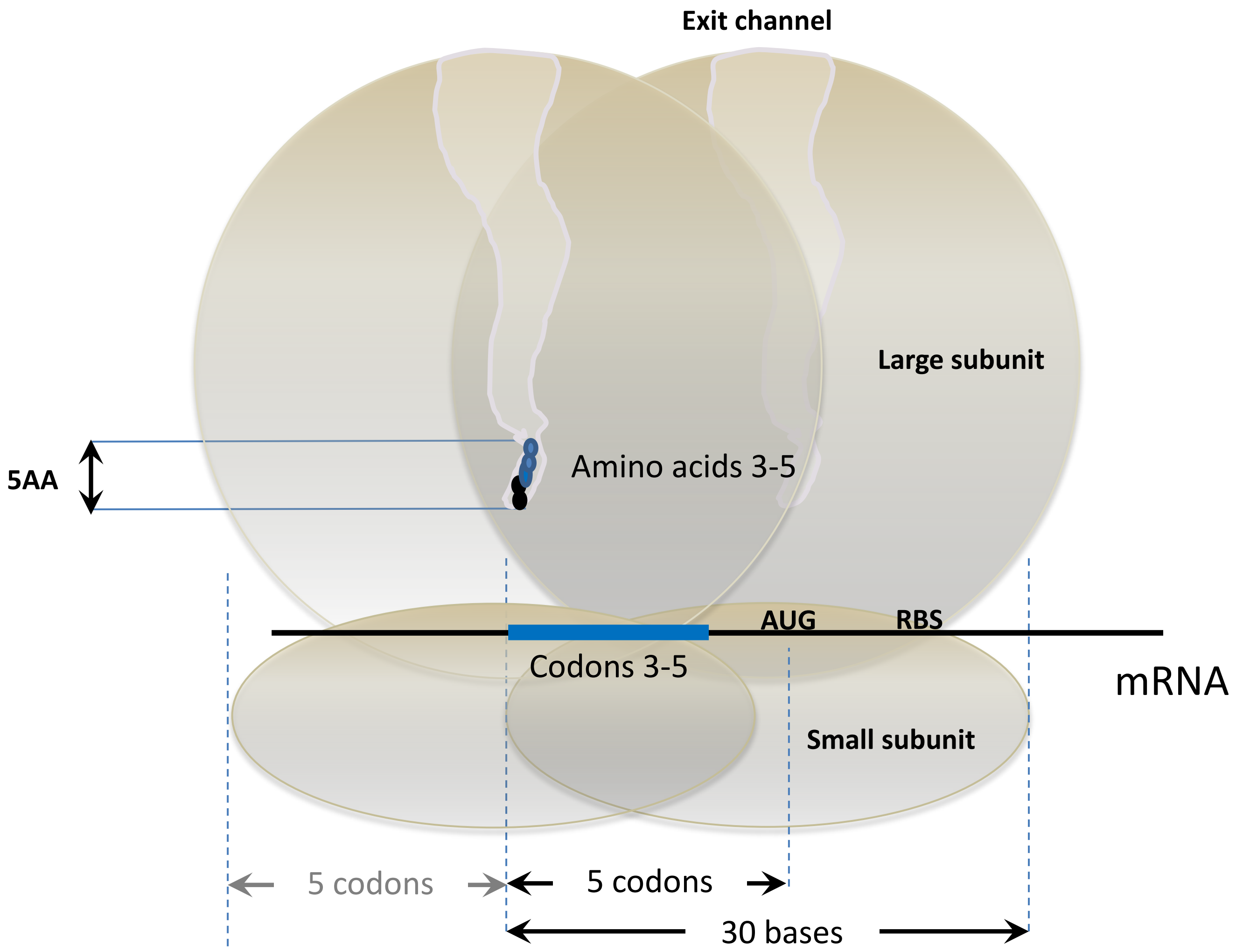

Supplementary Figure 2 | Scheme representing the first ribosome footprint and movement during the translation of the "short", five amino acid long (5AA), translational ramp described in this manuscript. Start codon (AUG), ribosome binding site (RBS), ribosomal subunits, ribosome footprint and first five amino acids in the peptide exit channel are indicated. Position of codons and amino acids 3-5 in ribosome is indicated in blue color.

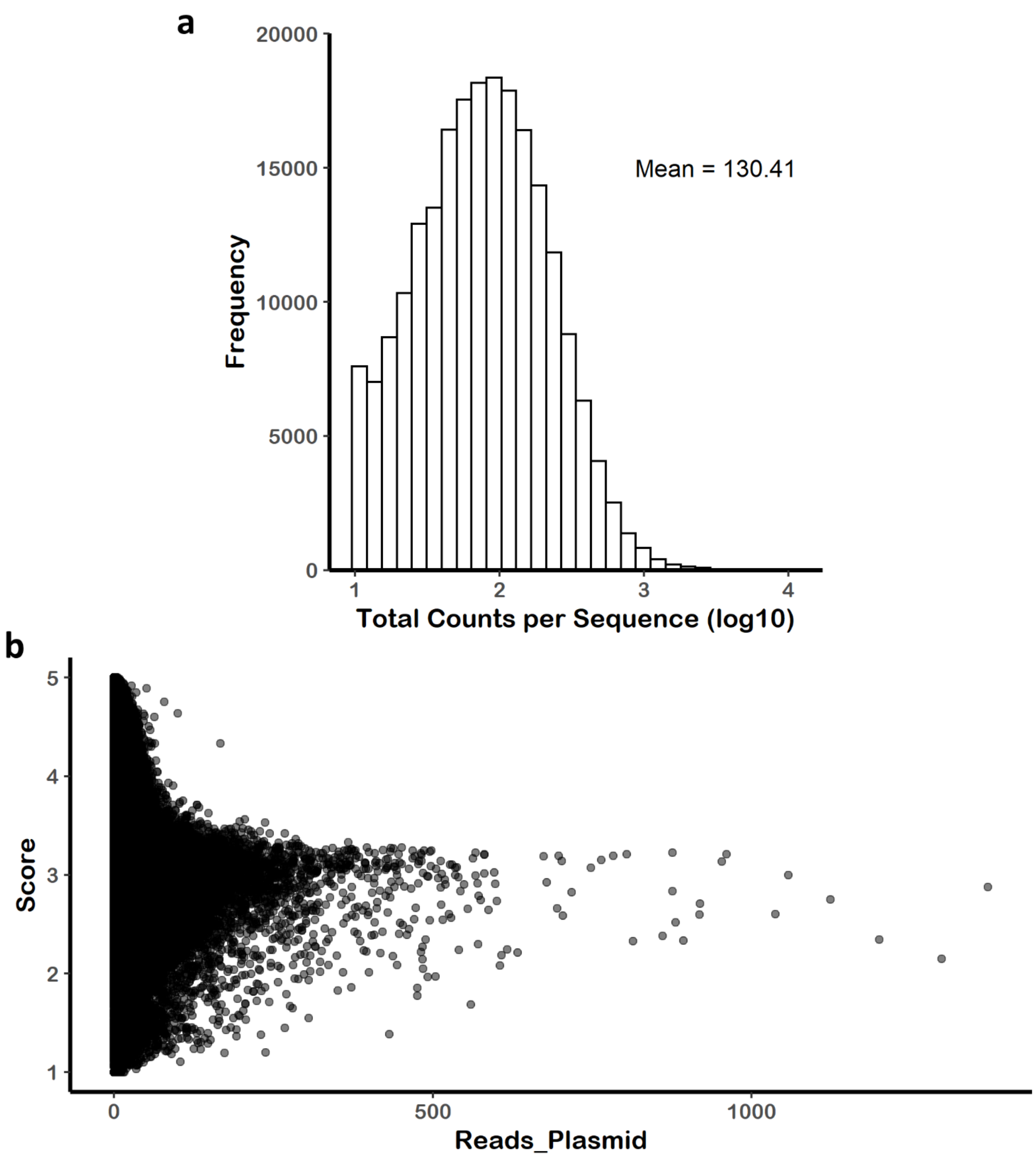

Supplementary Figure 3 | Descriptive plots for the eGFP reporter library. a Histogram of counts for each unique reporter construct across all five FACS sorted bins. **b** Scatter plot showing no correlation between the abundance of the reporter in the plasmid pool (Reads_Plasmid) and GFP Score (Score).

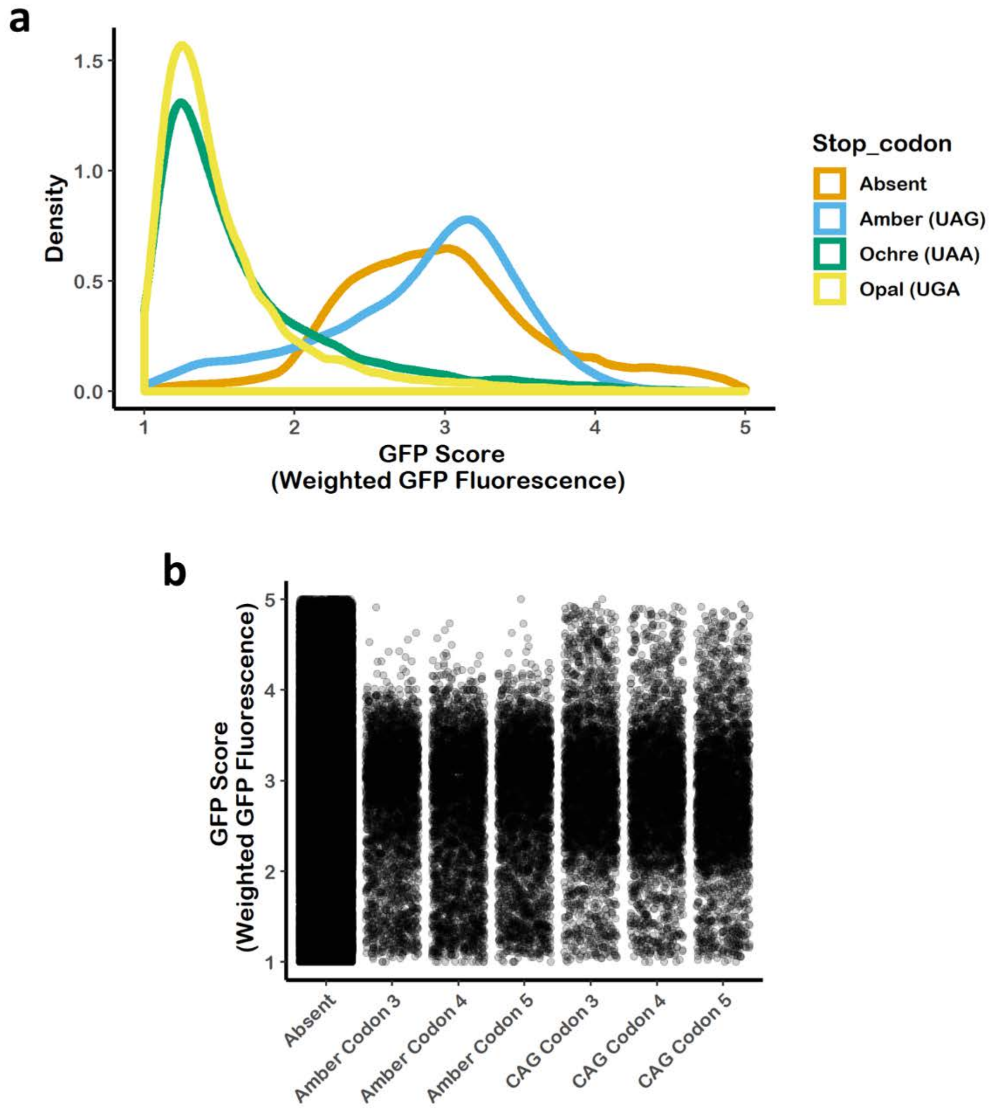

Supplementary Figure 4 | The effect of stop codon and rare codons on GFP Score. **a** Density plot of GFP Score for all reporter constructs lacking a stop codon (Absent) or with one or more Amber, Ochre or Opal stop codons. **b** Jitter plot of GFP Score for all constructs lacking an Amber stop codon or a Gln^{CAG} codon and constructs containing either Amber or Gln^{CAG} at position 1, 2 or 3 within the variable region (codons 4, 5 and 6 overall).

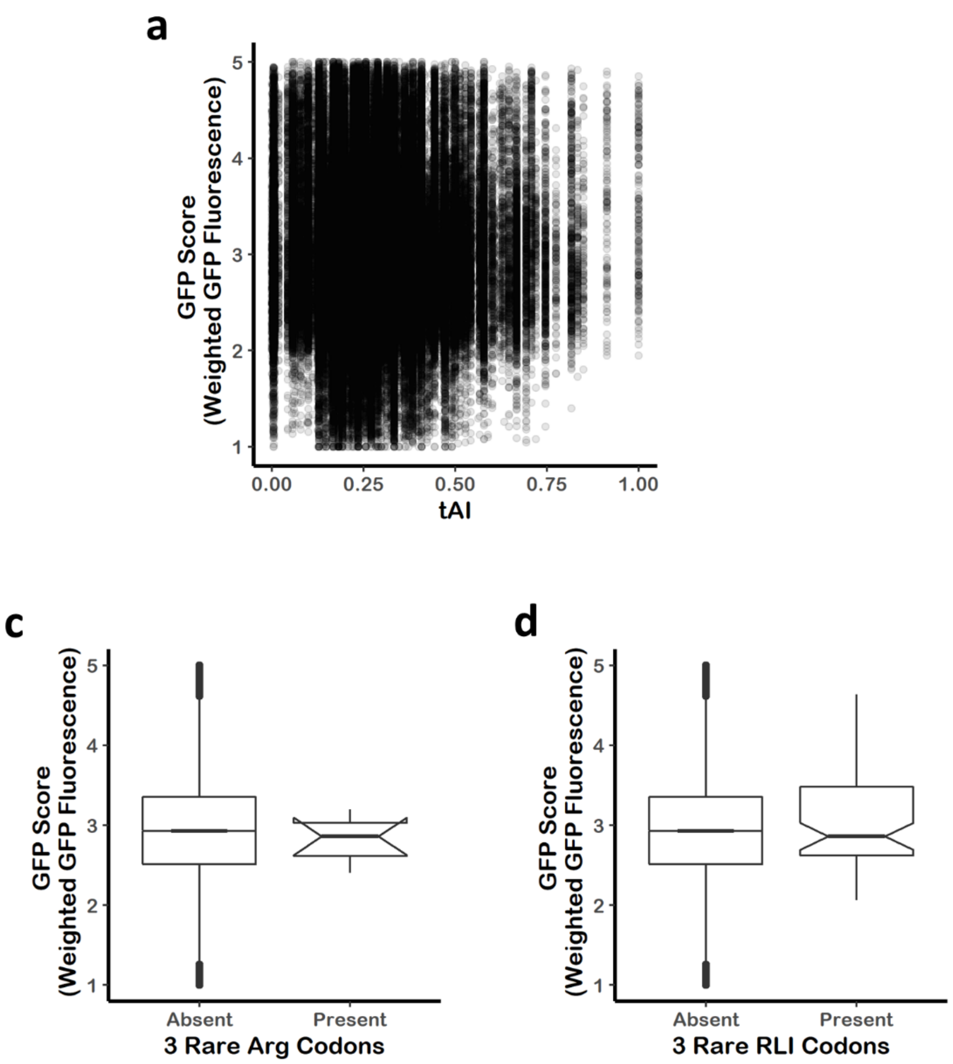

Supplementary Figure 5 | The effect of tRNA usage on protein GFP Score. **a** No correlation is observed between tRNA abundance (tAI –tRNA adaptation index) and the expression of eGFP variants based on GFP scores. **b** Box plot showing GFP Score for all reporters or those containing rare codons for R, L or I or R. **c** box plot showing GFP Score for reporters containing three rare arginine codons

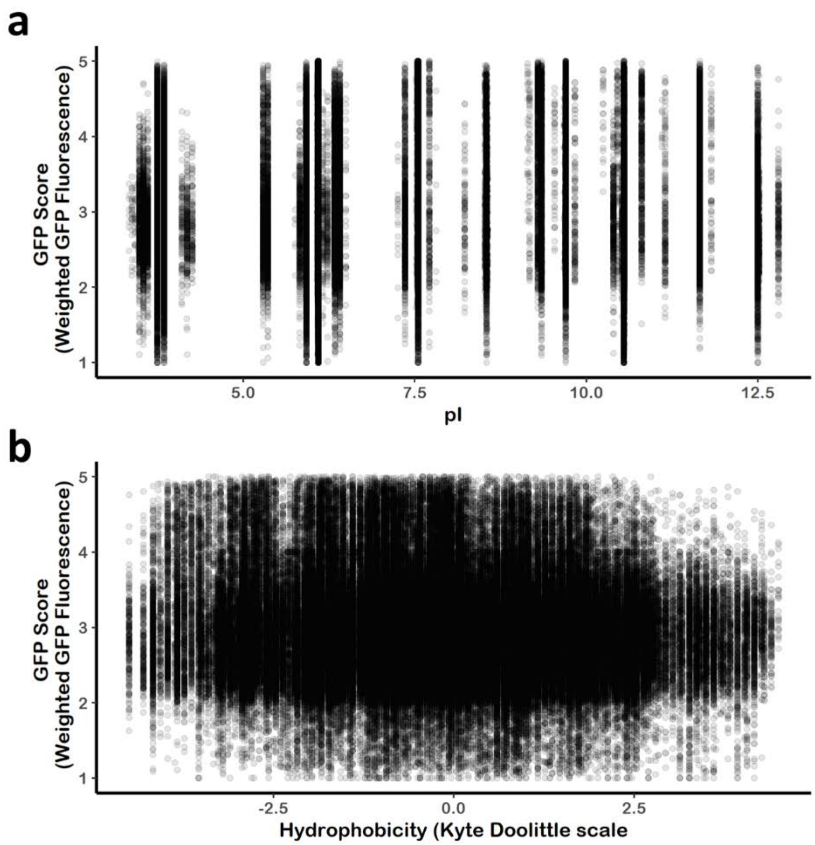

Supplementary Figure 6 | The effective of tri-peptide characteristics on GFP Score. a Scatter plot of GFP Score and the isoelectric point of the tri-peptide produced by the variable region within our GFP reporter library. Isoelectric point was determined by the R package Peptides using the EMBOSS pK scale. **b** Scatter plot of GFP Score and the hydrophobicity of the tri-peptide produced by the variable region within our GFP reporter library. Hydrophobicity was determined by the R package Peptides using the Kyte-Doolittle scale.

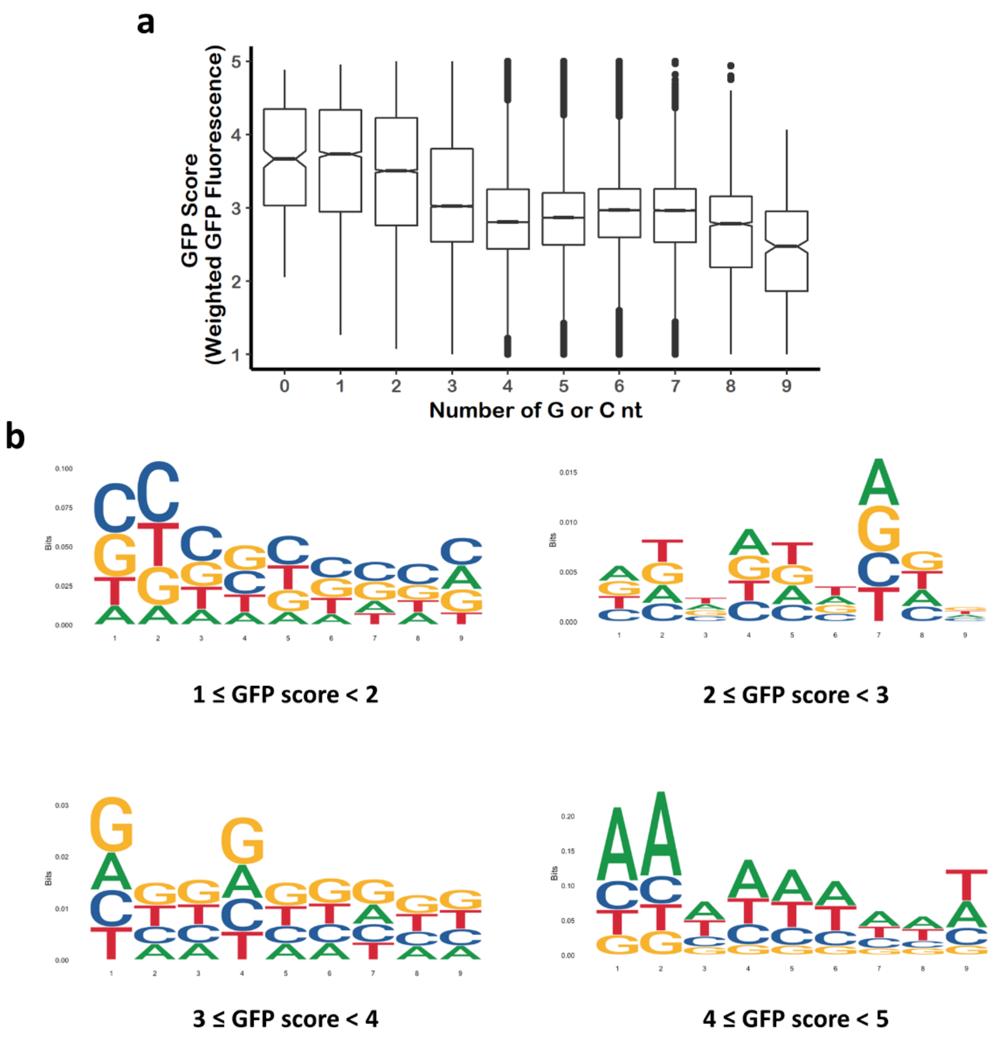

Supplementary Figure 7 | The effect of nucleotide content on GFP Score. a Box plot of GFP Score for all eGFP reporters within our library binned by the number of G or C nucleotides in the variable region. **b** Sequence logos for eGFP variants with defined GFP scores. Sequence logos were created from a collection of aligned sequences and depicts the consensus sequence and diversity of the sequences with each indicated score.

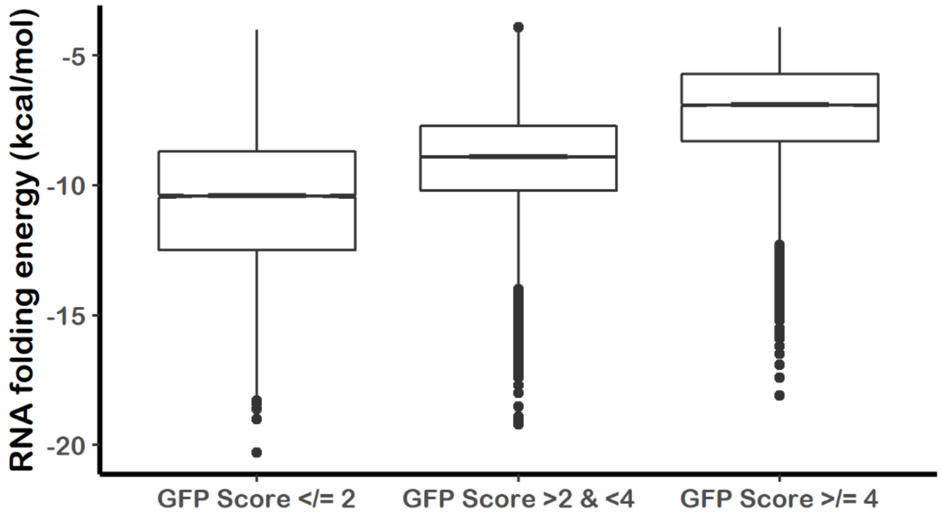

Supplementary Figure 8 | The effect of RNA structure GFP Score. Reporters are binned by GFP Score and RNA folding energy over variable region +/- 30 nt is shown.

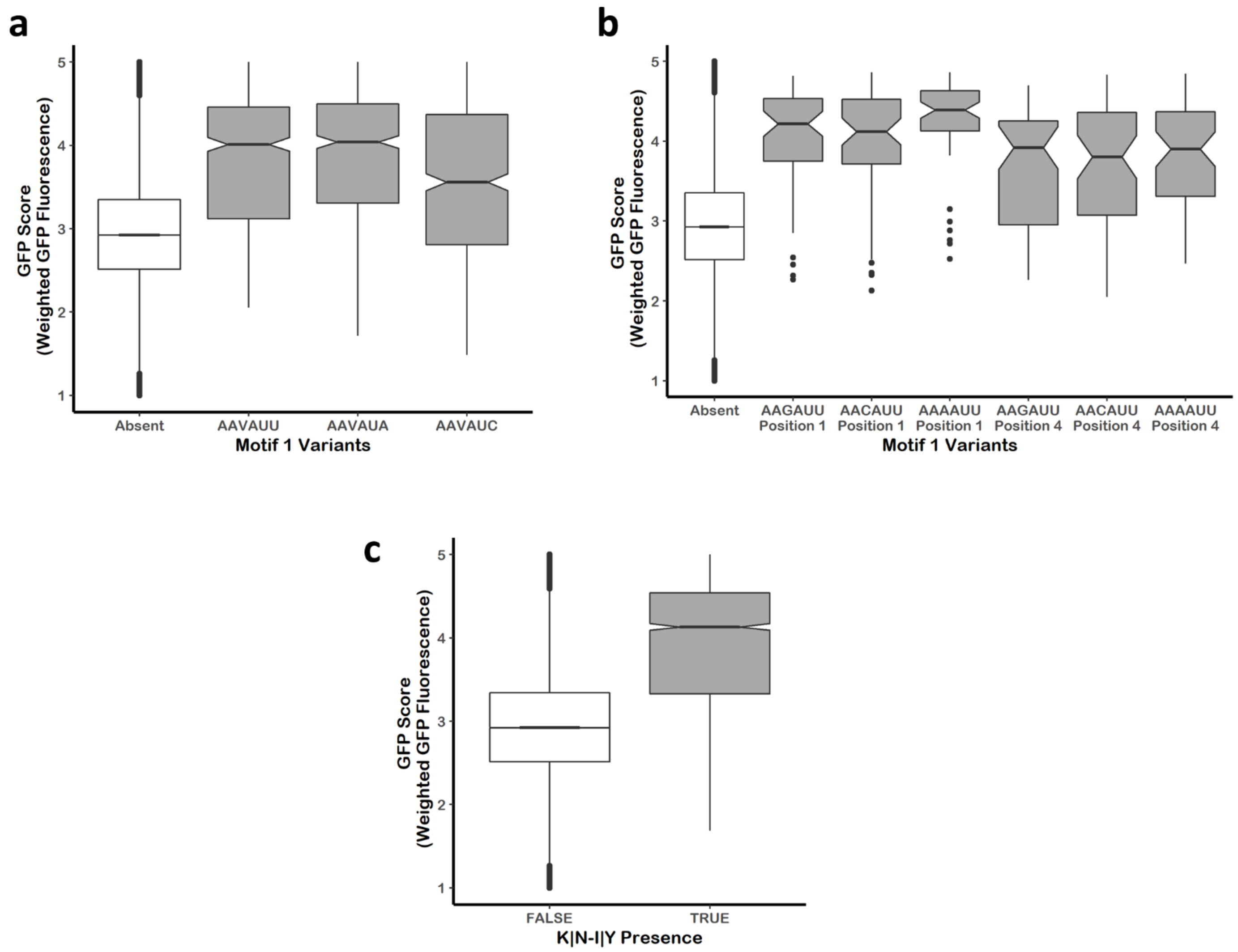

Supplementary Figure 9 | The effect of motif variants on GFP Score. a Boxplots exploring the effect of degenerate sequences of motif 1 (AAVAUU) on GFP Score. **b** Boxplots comparing variants of motif 1 starting at position 1 or 4 within the variable region of the eGFP reporter. **c** Boxplots comparing eGFP reporters containing the dipeptide K or N – I or Y (KI, KY, NI, NY) within the variable region or those reporters lacking the dipeptide.

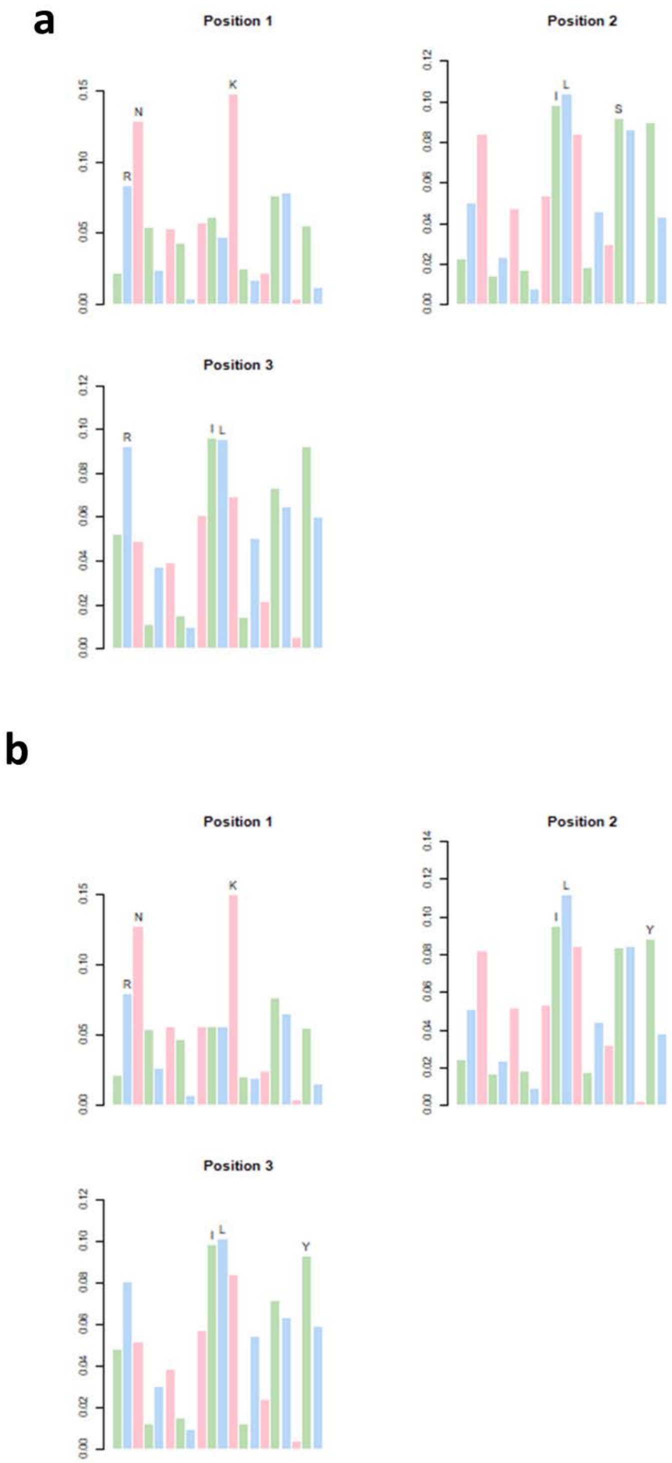

Supplementary Figure 10 | Amino acid represntation in reporters with high GFP Score. Bar charts showing frequency (y-axis) of each amino acid residue within the variable region of the eGFP reporters within our library for those reporters with high-GFP Score (>4). Panel a shows results from experiment 1 and panel b shows results from experiment 2.

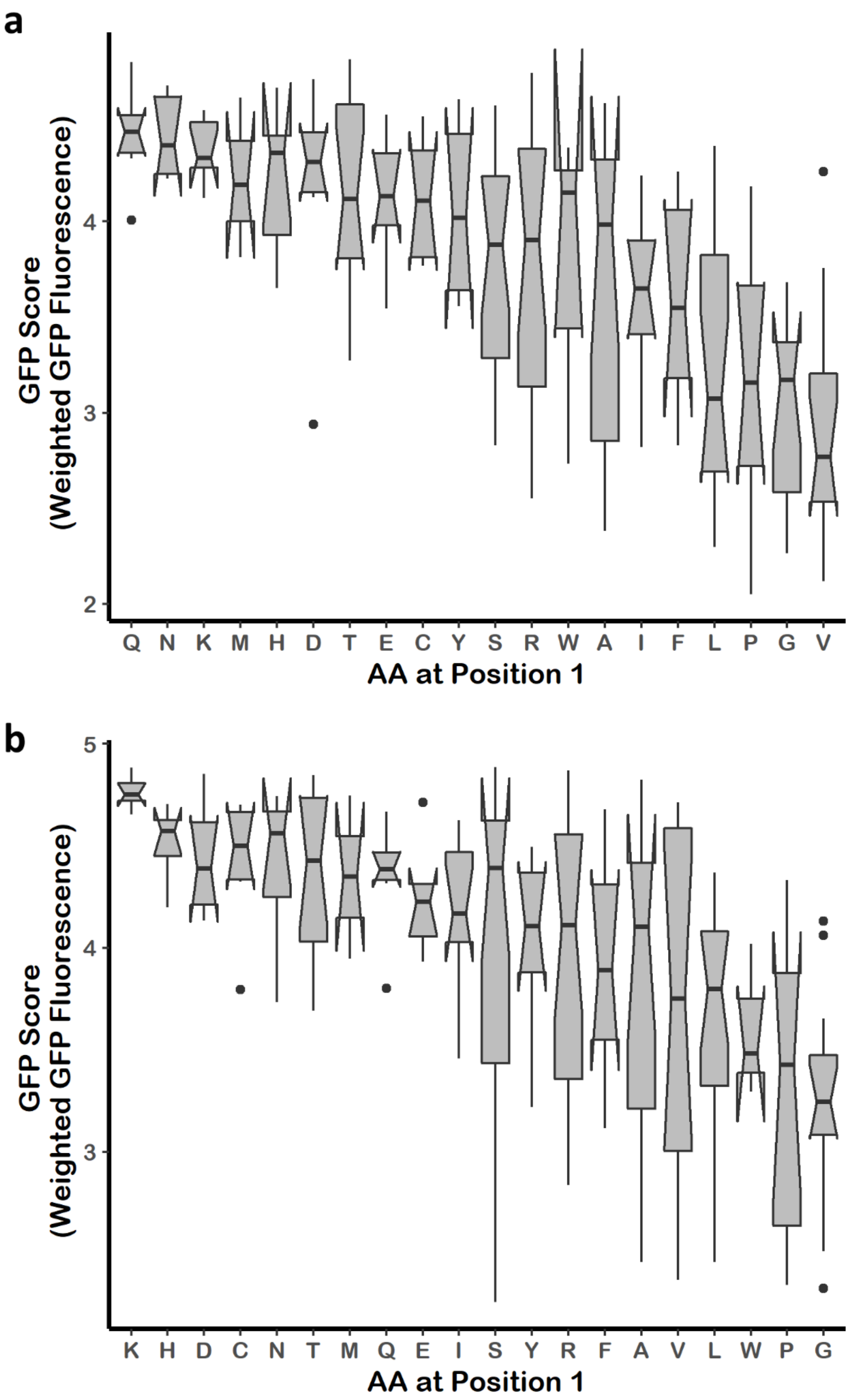

Supplementary Figure 11 | Interplay between nucleotide motifs and the first amino acid in the variable region. a Boxplots showing the effect of amino acid identity at position one for all reporters that contain motif 1 at position 4. **b** Boxplots showing the effect of amino acid identity at position one for all reporters that contain motif 2 at position 4.

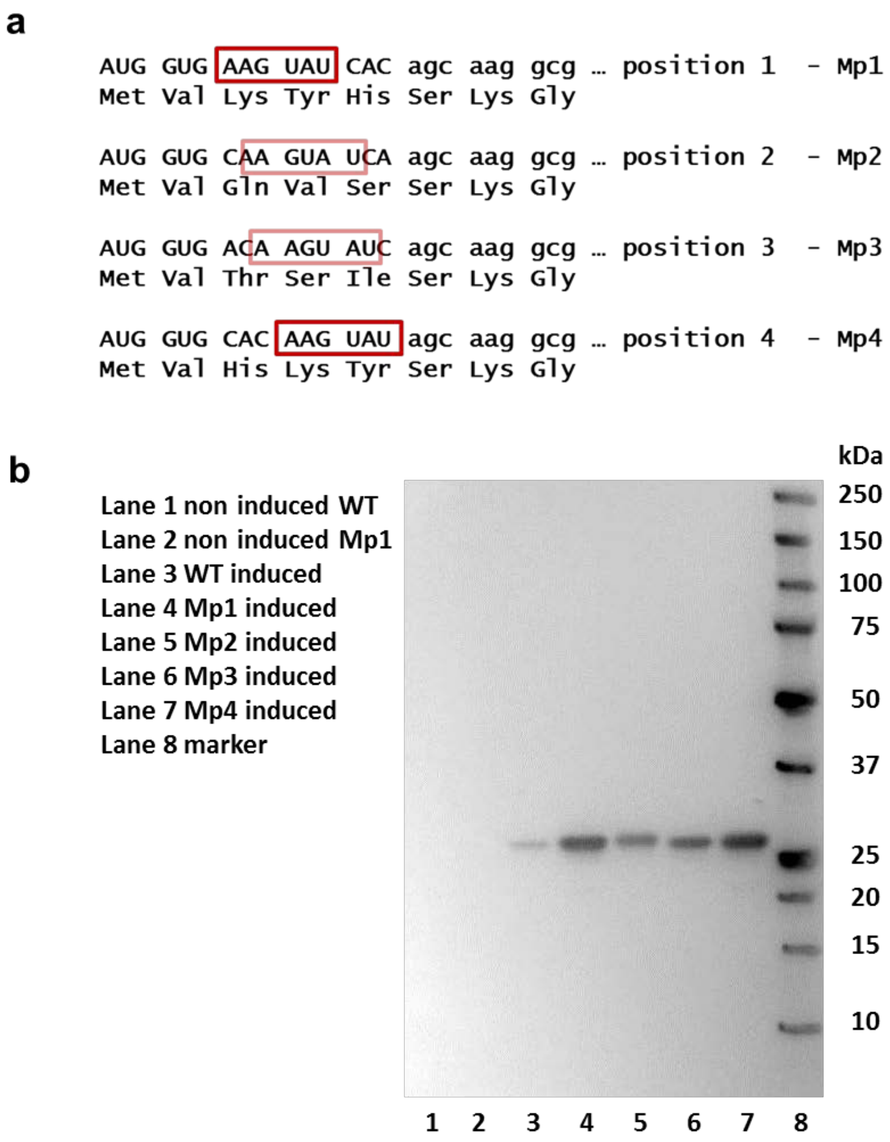

Supplementary Figure 12 | Expression of eGFP variants with Mp1-Mp4 motifs *in vivo* confirms FACS and bioinformatics analyses. a Scheme of analysis of the overall influence of amino acid sequence when motifs code for amino acids in positions 3 and 4 or 4 and 5, respectively. Mp1 indicates motif1 in position 1(ΔG=-8.50 kcal/mol, GFP score=4.47±0.57), Mp2 indicates motif1 in position 2 (ΔG=-8.00 kcal/mol, GFP score=3.93±0.68), Mp3 indicates motif1 in position 3 (ΔG=-7.7 kcal/mol, GFP score=3.59±0.78) and Mp4 indicates motif1 in position 4 (ΔG=-6.60 kcal/mol, GFP score=4.15±0.68). Sequence of each construct and position of the motif are indicated. **b.** Western-blot analysis of *in vivo* expression of eGFP constructs with motif AAG UAU in different positions coding for amino acids 3, 4 and 5. pBAD low copy vector in *E. coli* BL21 cells was used for *in vivo* expression. Equal number of E. coli cells (OD_{600}) following a 3 hour induction with 0.2% arabinose was used for western-blot analyses. Non-induced wild-type (WT, ΔG=-7.70 kcal/mol, GFP score=2.03±0.53) and Mp1 constructs are used as controls. eGFP antibody (JL-8, Clontech) and anti-mouse HRP-conjugated secondary antibody were used to visualize expression of eGFP. Biorad Precision Plus marker is indicated in image.

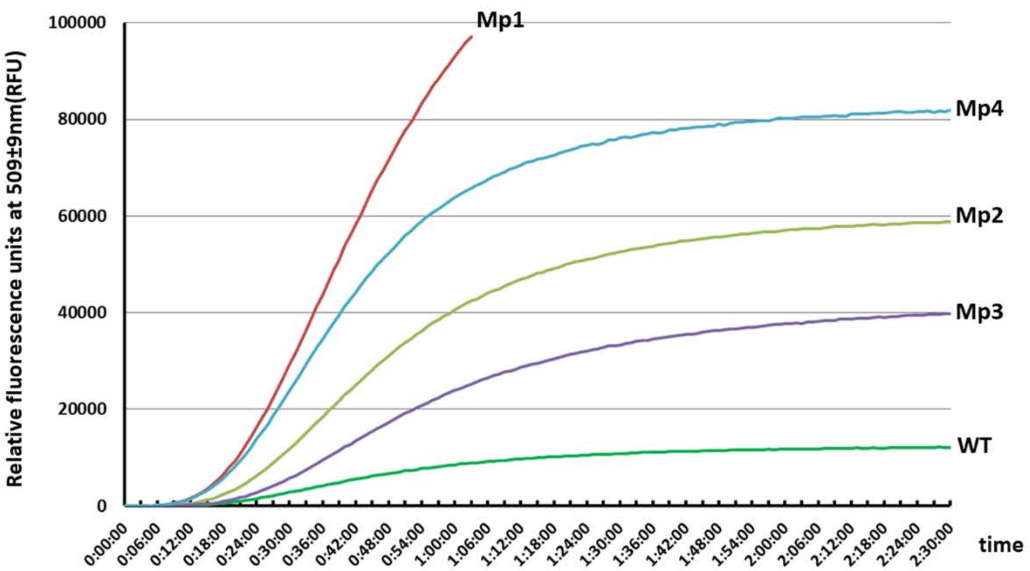

Supplementary Figure 13 |Kinetics of of NEB Pure Express *in vitro* expression of eGFP constructs. The expression of eGFP variants was followed by relative fluorescence (RFU) emission at 509 ± 9nm. 100 ng of Mp1-Mp4 and WT eGFP varaints from Figure 3A-B and Supplementary Figure 9a were used as templates for in vitro reaction.

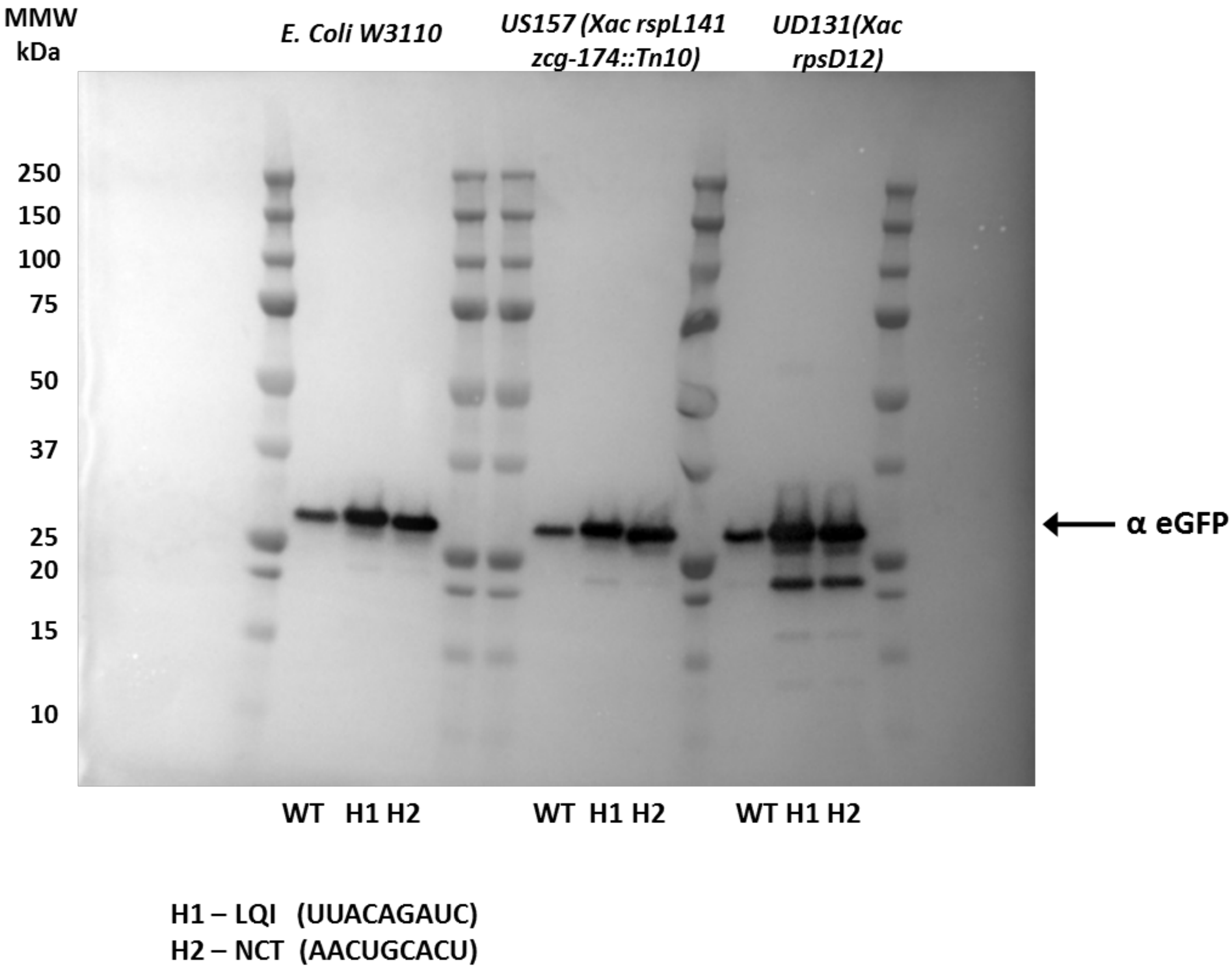

Supplementary Figure 14 | Western-blot analysis of expression of eGFP construct variants in different E. coli strains. eGFP variants were transfected and expressed from pBAD low copy vector in *E. coli* W3110, US157 and UD131 strains. Two high expression variants H1 (NCT) and H2 (LQI) and WT eGFP constructs are indicated. Nucleotides in position 7-15 are indicated for two high expressing variants. Equal number of E. coli cell (OD_{600}) was used for western blot analyses after 3 hour induction period with 0.2% arabinose. eGFP antibody (JL-8, Clontech) and anti-mouse HRP-conjugated secondary antibody were used to visualize expression of eGFP. Biorad Precision Plus marker is indicated in image.

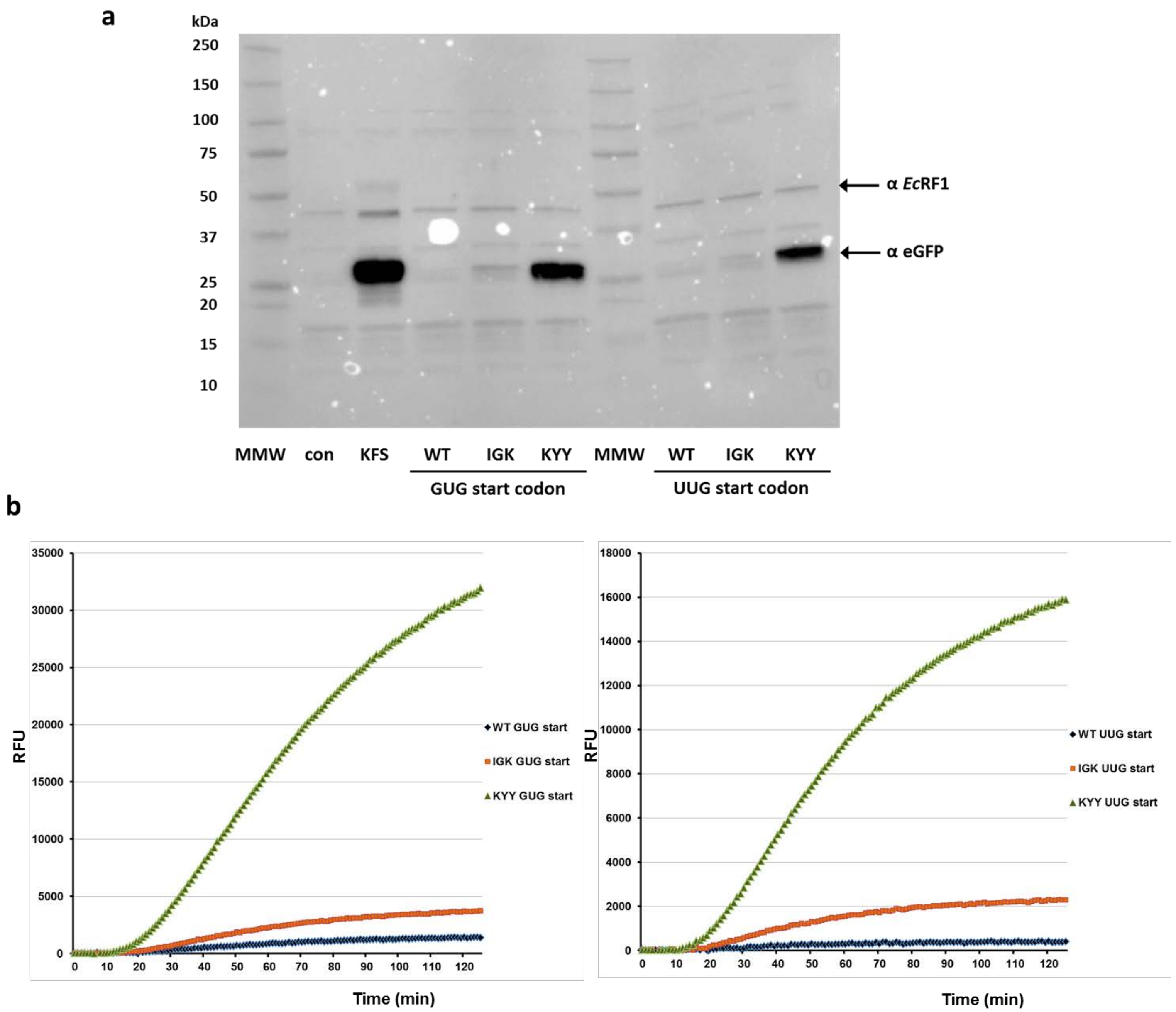

Supplementary Figure 15 | Alternative start codons show same difference in expression of eGFP variants. **a** Western blot analysis of NEB Pure Express *in vitro* expression of eGFP constructs with near cognate start codons GUG and UUG. eGFP construct with AUG start codon and KFS peptide in amino acid position 3-5 is indicated. 100ng of each PCR product with T7 promoter seuqnce was used for *in vitro* expression. Control (con) is NEB Pure Express *in vitro* expression reaction mix without template. 5% of in vitro translation reaction is analyzed. IGK (GFP score 3.00±0.56; ΔG=-8.40 kcal/mol) and KYY (GFP score 4.84±0.15; ΔG=-8.00 kcal/mol) constructs indicate amino acids in positions 3-5 of eGFP variants. eGFP antibody (JL-8, Clontech), E. coli peptide release factor I (αEcRF1), anti-mouse and anti-rabbit HRP-conjugated secondary antibodies were used to visualize expression of eGFP and normalization of western blot data, respectively. Biorad Precision Plus marker is indicated in image. **b** The expression of eGFP variants with near start GUG and UUG codons were followed by relative fluorescence (RFU) emission at 509 ± 9nm. 100 ng of of each construct were used as templates for in vitro reaction using NEB Pure Express in vitro expression system.

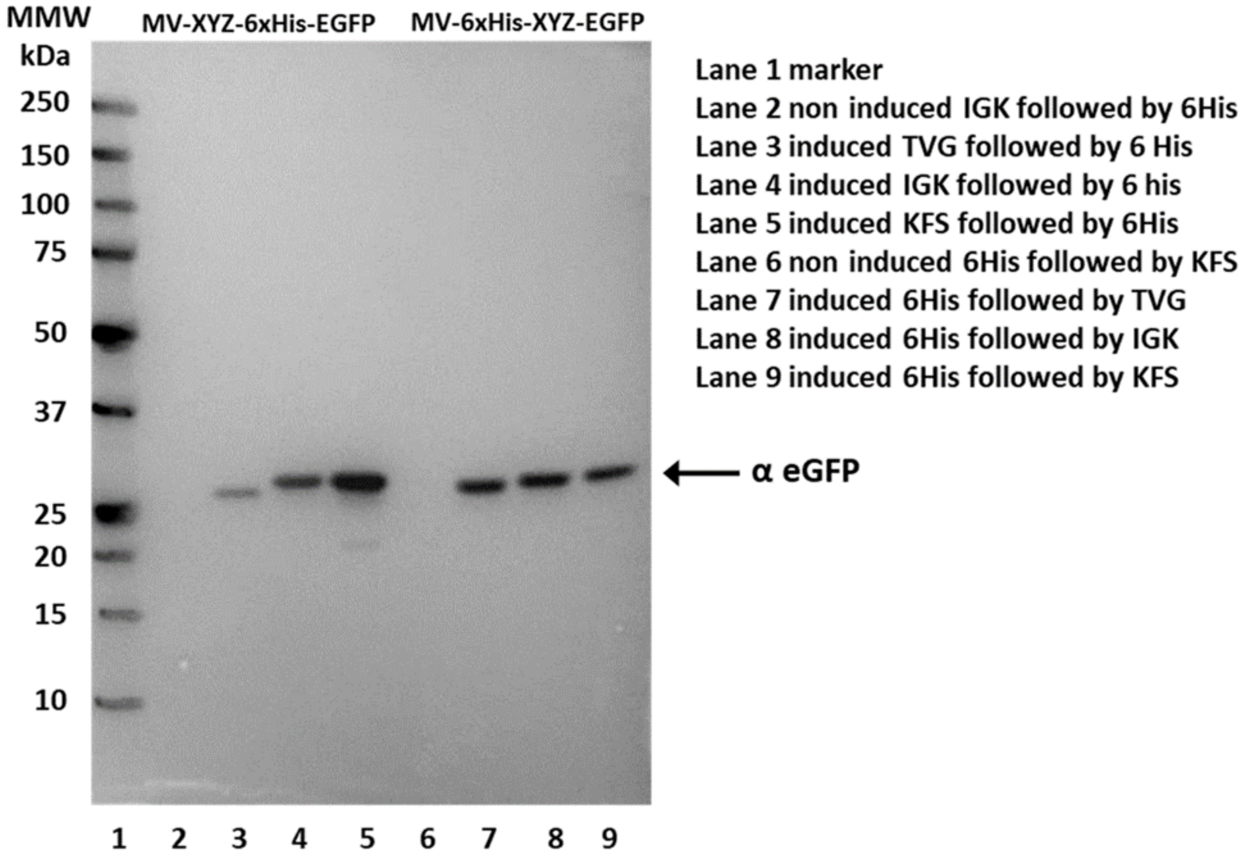

Supplementary Figure 16 | Positional bias in controlling expression of eGFP constructs with different amino acids in position 3, 4 and 5. Western-blot analysis of *in vivo* expression of eGFP constructs with sequence XYZ (KFS, IGK and TVG, respectively) as amino acids 3(X), 4(Y) and 5(Z) followed by 6xHis tag (MV-XYZ-6xHis) or as amino acids 9(X), 10(Y) and 11(Z) preceded by 6xHis tag (MV-6xHis-XYZ). *E. coli* BL21 cells were used for expression and equal number of cells (OD_{600}) was used for analyses upon 3 hour induction period with 0.2% arabinose. eGFP antibody (JL-8, Clontech) and anti-mouse HRP-conjugated secondary antibody were used to visualize expression of eGFP. Biorad Precision Plus marker is indicated in image.

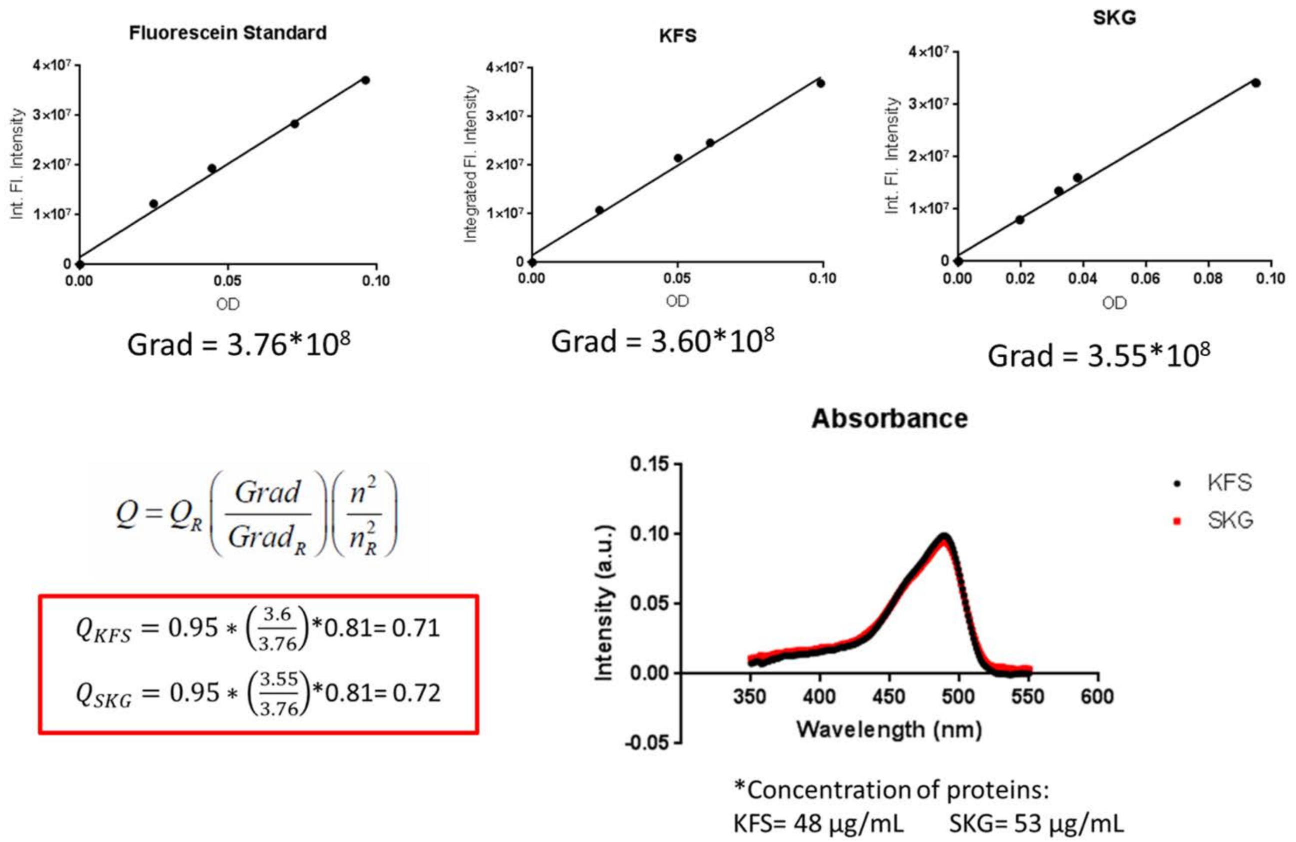

Supplementary Figure 17| Normalized absorbance spectrum and quantum yield calculation for the KFS eGFP variant and wt SKG eGFP. Absorption spectra were background corrected for solvent (PBS) absorbance (lower right), and the optical density was determined as a function of excitation wavelength, in a range of values from 0.01 to 0.1. Quantum yield has been calculated as relative quantum yield compared to a fluorescein standard (Q=0.95, measured at 22C in NaOH). The area under the curve of background-subtracted fluorescent emission spectra were calculated (Vinci ISS software) to obtain integrated fluorescence intensities (top row). The gradient (slope) values of integrated fluorescence intensity vs. optical density were linearly fit and used for the calculation of the quantum yields (red box).

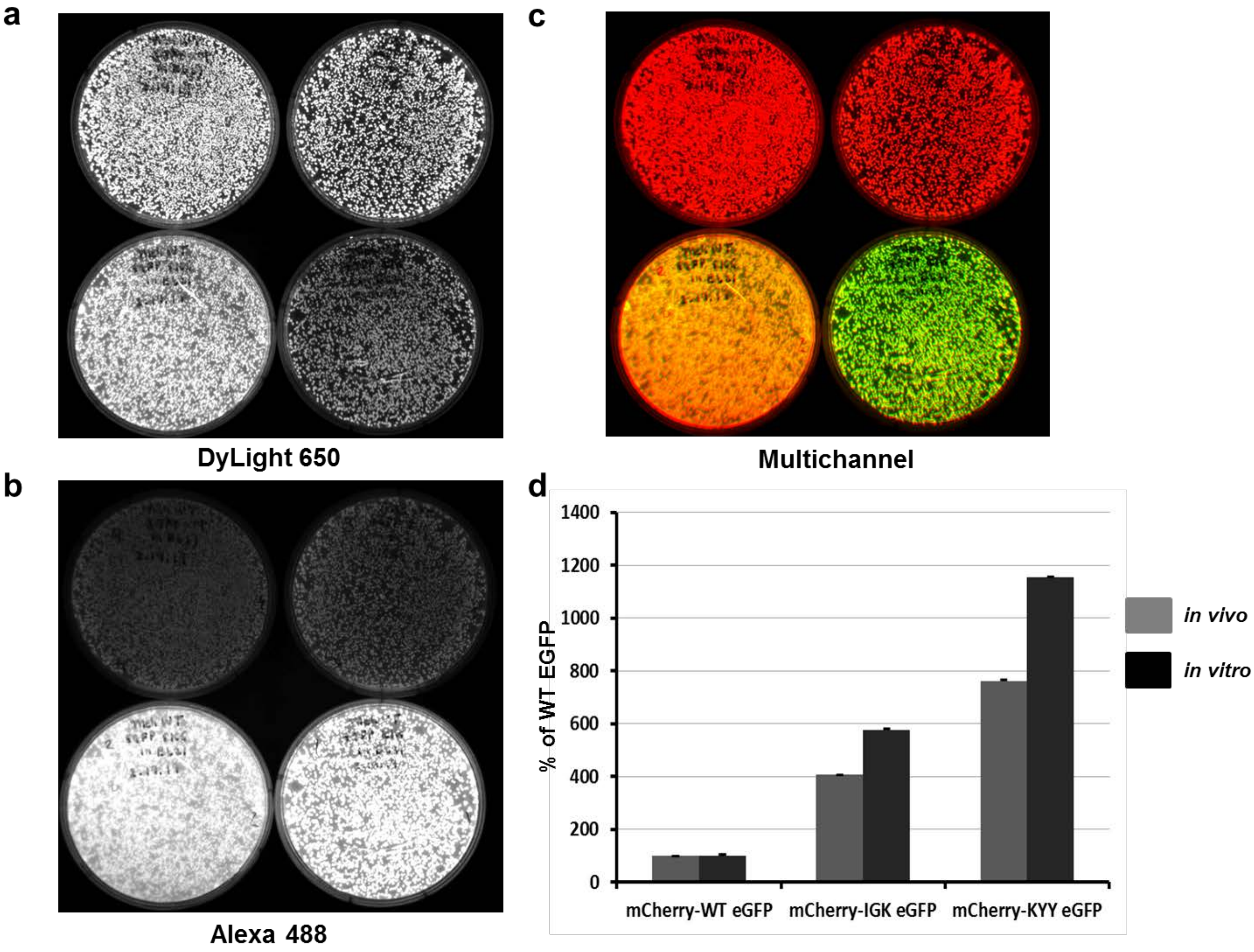

Supplementary Figure 18 | Polycistronic expression of eGFP constructs with different amino acids in position 3, 4 and 5 as a second ORF from petDUET vector. a,b,c Images of *E. coli* colonies on the LB-agar plates (Carb 100ug/ml, 0.2% Arabinose) expresssing mCherry-eGFP polycistronic constructs from petDUET vector (Novagen). Wild type (WT; ΔG=-7.90 kcal/mol) or eGFP constructs with sequence KYY (ΔG=-7.00 kcal/mol), IGK (ΔG=-11.40 kcal/mol) and TVG (ΔG=-16.00 kcal/mol) as amino acids inserted in position 3,4 and 5 are shown. Dylight 650 (**a**), Alexa488 (**b**) or both filters (**c**) were used to image expression of mCherry or eGFP proteins, respectively. **d** Normalized ratio of eGFP *in vivo* and *in vitro* expression using polycistronic mCherry-eGFP constructs from petDUET vector. *E. coli BL21* cells were used for *in vivo* and NEB Pure Express for *in vitro* expression. Cells were induced for 3hours with 0.2% arabinose and fluorescence is measured 3 hours after induction. 100ng of each PCR product with T7 promoter seuqnce was used for *in vitro* expression. Data for eGFP fluorescence were normalized by mCherry fluorescence and represented compared to WT eGFP construct.

```
hRGS2    MQSAMFLAVQHDCRPMDKSAGSGHKSEEKREKMKRTLLKDWKTR...
M1       MVQSALFLAVQHDCRPLDKSAGSGHKSEEKREKLKRTLLKDWKT...          (CAAAGUGCU)
M5       MVLAVQHDCRPLDKSAGSGHKSEEKREKLKRTLLKDWKTRLSYR...          (UUGGCUGUU)
M16      MVKSAGSGHKSEEKREKLKRTLLKDWKTRLSYRLSYFLQNSSTR...          (AAGAGCGCA)
M33      MVRTLLKDWKTRLSYRLSYFLQNSSTRLSYFLQNSSTPGKPKTG...          (CGGACUCUU)
```

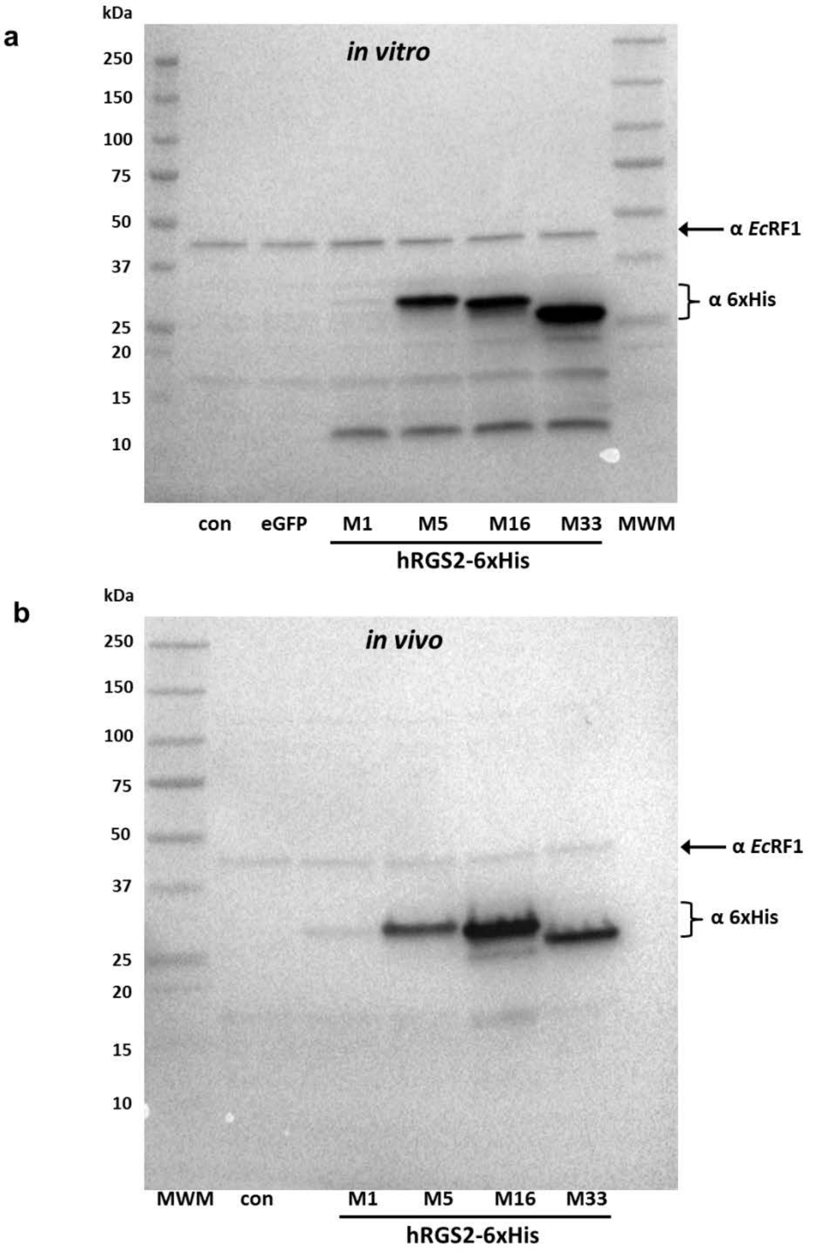

Supplementary Figure 19| Expression of recombinant human protein with alternative start sites in NEB PURE *in vitro* expression system (a) or *in vivo* in *E. coli* cells (b) follows previously obtained GFP score for eGFP varaints. Scheme and sequences of wild type human RGS2 protein (hRGS2) and four tested constructs with alternative starting methionine (M1, M5, M16 and M33) are indicated. Alternative start site methionine (M) residues are mutated into leucine residues (L) to maintain one methionine residue per construct. **a & b** Western blot analyses of NEB Pure Express *in vitro* or *in vivo* *E. coli* expression of hRGS2 variants as well controls (con and eGFP) are indicated. 100ng of each hRGS2 variant with T7 promoter sequence was used in *in vitro* reaction. *In vivo* expression was assayed 3 hours after induction (0.2% arabinose) of the same amount of cells (OD_{600}). Proteins were visualized based on their C-terminal 6-Hist tag using Penta-His (Qiagen) antibody. E. coli peptide release factor I (αEcRF1) and anti-rabbit HRP-conjugated secondary antibody was used for normalization of western blot analyses. Biorad Precision Plus marker is indicated in image.

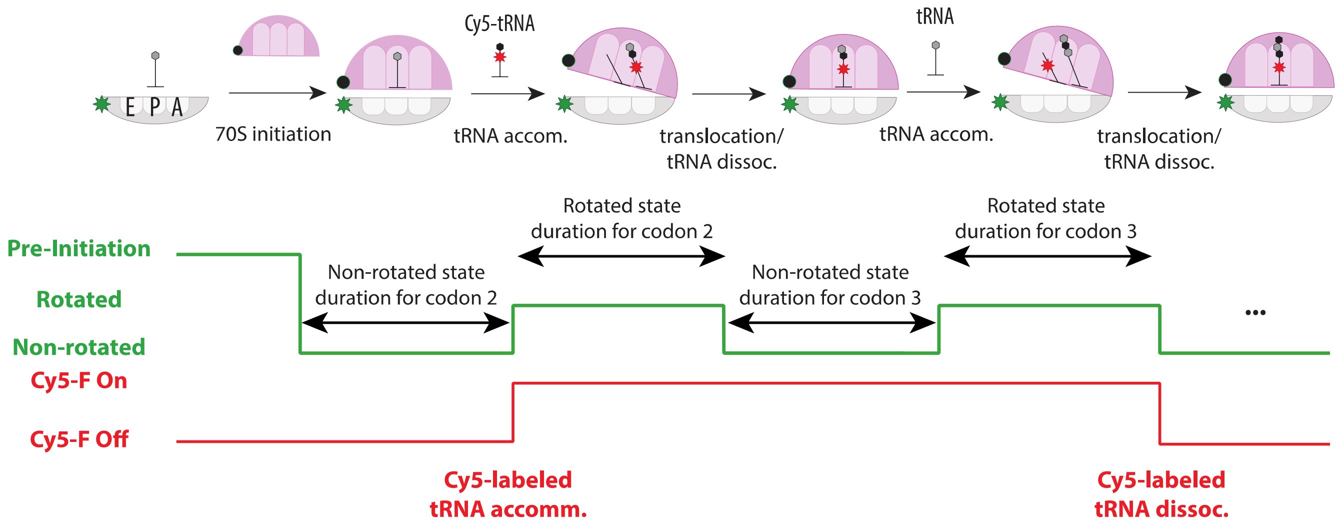

Supplementary Figure 20 | Progression of translation elongation and expected single-molecule fluorescence signal in each step. Prior to the experiment, Pre-Initiation Complex (PIC) composed of Cy3B-labeled small ribosomal subunit (Cy3B-30S; Cy3B denoted as a green star in the schematics), initiation factor 2 (IF2; not shown), initiator fMet tRNA and 5'-biotinylated mRNA (not shown) is formed and tethered to the SMRT cell (purchased from Pacific Biosciences) patterned with zero-mode waveguides. The SMRT cell is Neutravidin-coated to allow tethering of PIC via Biotin-Neutravidin interaction from the 5'-end of mRNA. In the beginning of the experiment, buffer containing elongation factors (including Phe-Cy5-tRNA^{Phe}; Cy5 denoted as red star in the schematics) and BHQ-2-labeled large ribosomal subunit (BHQ-50S; BHQ denoted as black circle in the schematics) are delivered to the SMRT cell. Upon initiation of translation, BHQ-50S binds to the 30S PIC and brings BHQ-2 close to Cy3B such that Cy3B fluorescence (denoted as green trace) is quenched by Förster Resonance Energy Transfer (FRET) at the non-rotated state. After 70S initiation step, cognate tRNA to codon presented in the A site (Phe codon in this case) can bind to the ribosome and accommodated for peptidyl-transferase reaction. Successful peptide-transfer is coupled with ribosome conformational change from non-rotated state to rotated state, where ribosomal subunits rotate respect to each other by about 7 degrees. Rotated state is a correct substrate for translocation catalyzed by elongation factor-G (EF-G; not shown), which moves the complex to the next codon and resets ribosomal conformation to non-rotated state, allowing next cycle of elongation to begin. Time between transitions are collected and fitted via single-exponential function to calculate non-rotated and rotated state lifetimes for codon present in the A site.

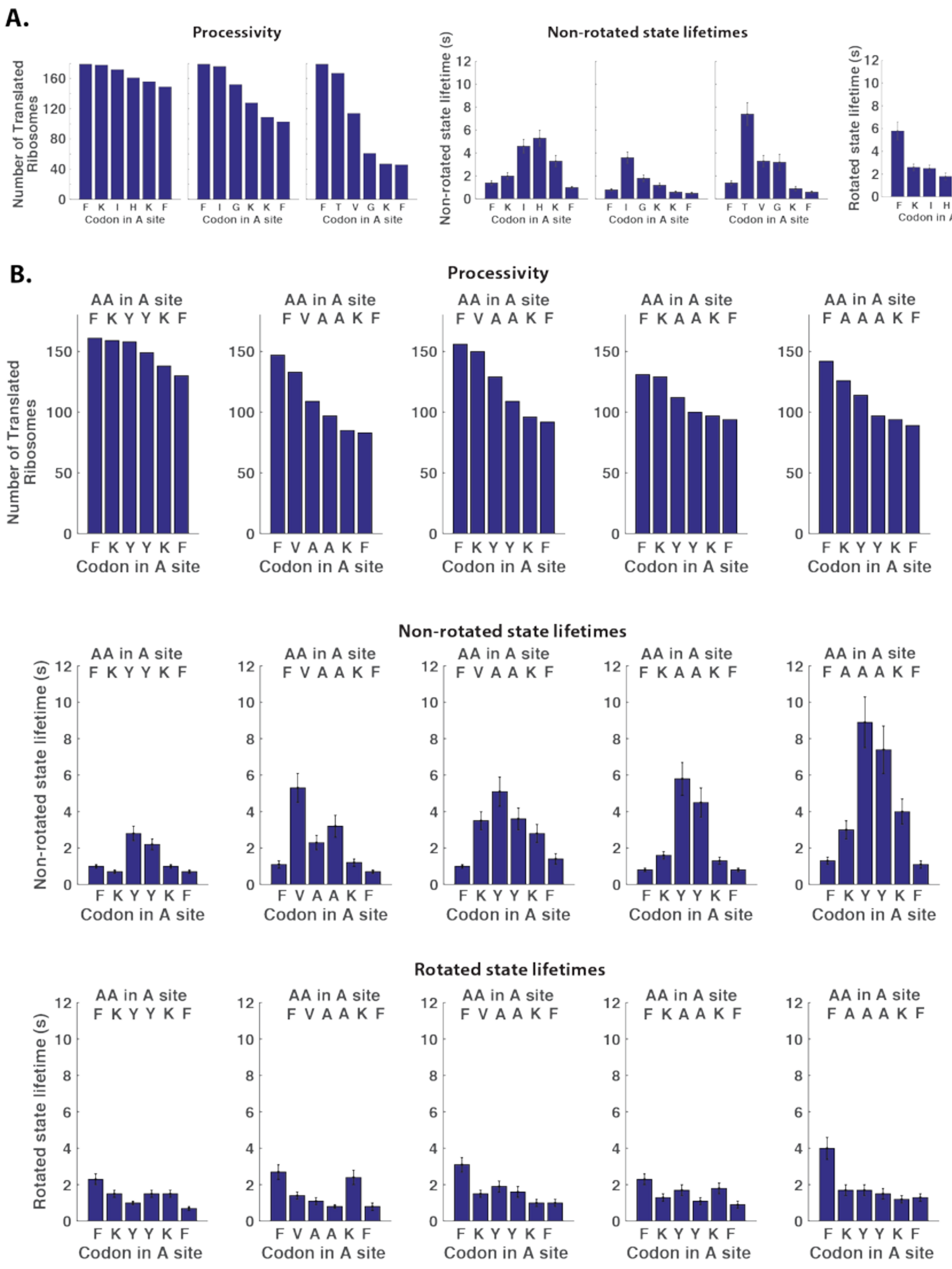

Supplementary Figure 21 | Processivity and state lifetime measured for data shown in Figure 7C and 8D. For each mRNA construct with different codon 3-5, the ribosome processivity (counted number of ribosome translated the particular codon) as well as non-rotated and rotated state lifetimes were measured for each codons (from fluorescence signals described in the **Supplementary Figure 20**). **A.** Processivity and state lifetime measured for construct shown in **Figure 7C**. **B.** Processivity and state lifetime measured for construct shown in **Figure 8D**. Percentage of molecules translating the entire ORF is calculated as percentage of ribosomes that have translated the last codon within the ORF (F_7 in this case) of the ribosomes that have translated the first codon after the start codon (F_2 in this case). Error bars for state lifetimes are 95% confidence intervals from fitting the single-exponential distribution.

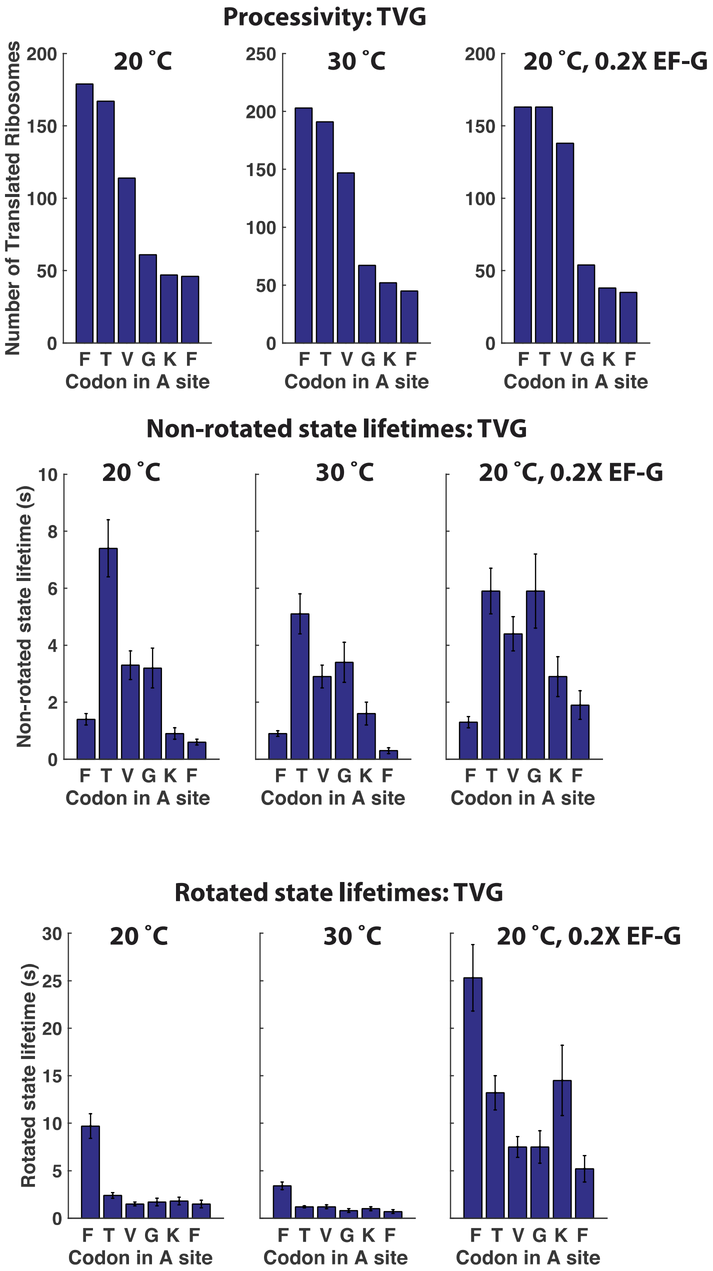

Supplementary Figure 22 | Processivity and state lifetime measured for $T_3V_4G_5$ construct at different experimental condition. For $T_3V_4G_5$ mRNA construct with different codon 3-5, the ribosome processivity as well as non-rotated and rotated state lifetimes were measured for each codons (as described in the **Supplementary Figure 20**) at three different conditions: at 20 °C (same as shown in the **Supplementary Figure 21**), at 30 °C, and at 20 °C with 5-fold less concentration of elongation factor-G (EF-G). The processivity shown here at top is used to calculated the percentage of molecules translating the entire ORF: 26 ± 3 % (at 20 °C), 22 ± 3% (at 30 °C) and 21 ± 3% (at 20 °C with 5-fold less EF-G; 20 nM). Lowering EF-G concentration increases the rotated state lifetimes for all codons, as they are rate-limited by EF-G association kinetics. Error bars for state lifetimes are 95% confidence intervals from fitting the single-exponential distribution.

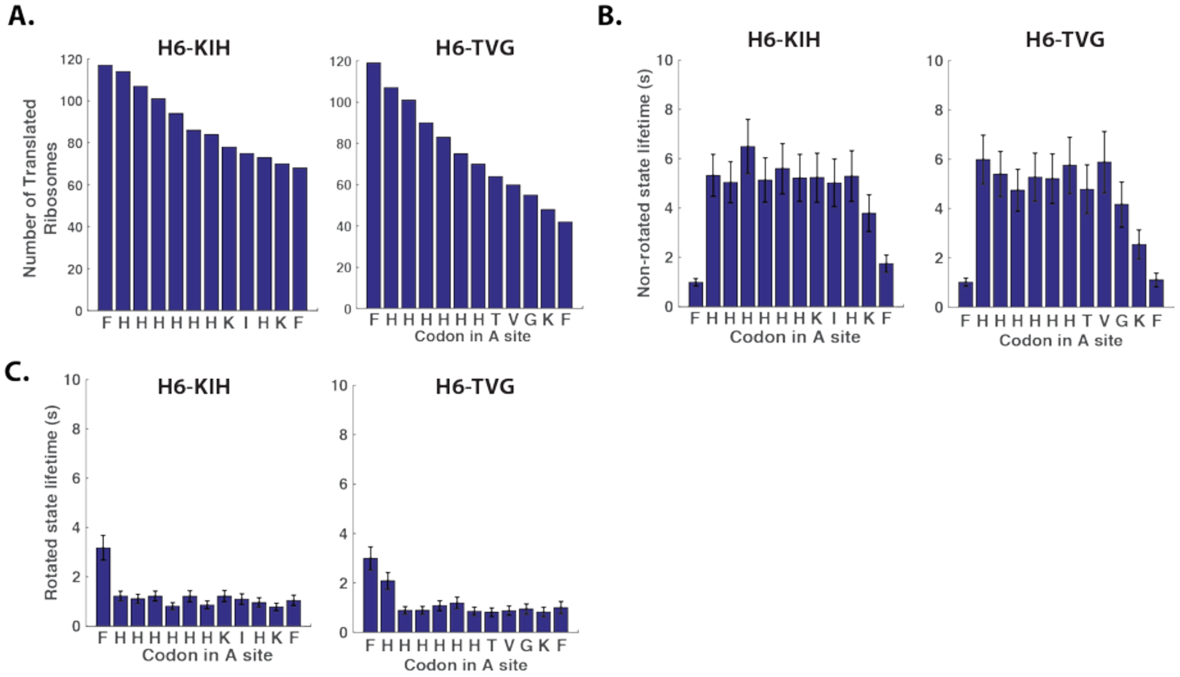

Supplementary Figure 23 | Positional bias in controlling ribosome processivity with different amino acids in position 3, 4 and 5. Similar to shown in **Supplementary Figure 16**, 6xHis tag sequences are added for codon position 3-8 before amino acids 9(X), 10(Y) and 11(Z). **A-C** Processivity (**A**) and state lifetime (Non-rotated in **B** and Rotated in **C**) measured for two constructs (H6-KIH and H6-TVG, encoding for peptides MFHHHHHHKIHKF and MFHHHHHHTVGKF). Error bars for state lifetimes are 95% confidence intervals from fitting the single-exponential distribution.

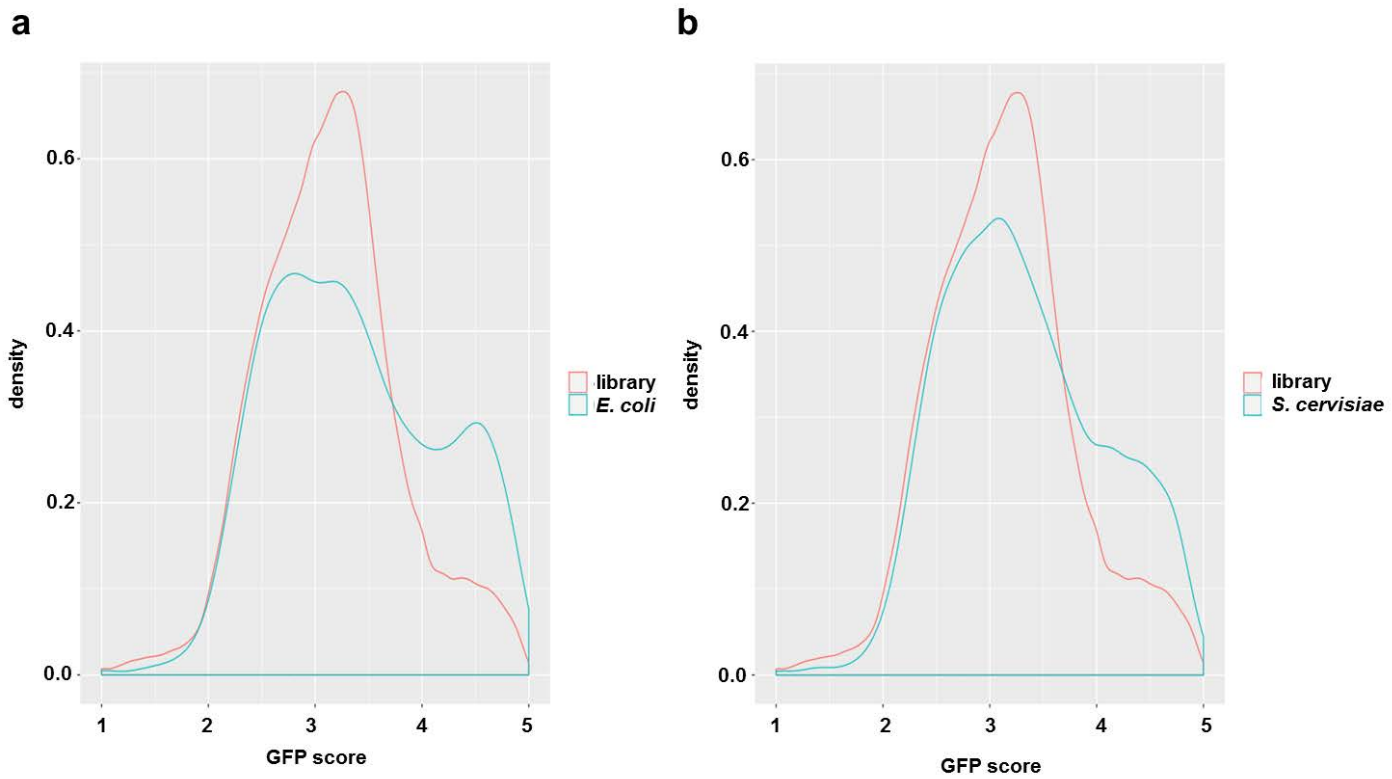

Supplementary Figure 24 | Comparison of GFP scores for eGFP library and natural sequences reveals transcriptome optimization in *E. coli* and *S. cerevisiae.* a and b *E. coli* and S. cervisiae endogenous transcripts (from genome builds ASM80076v1 and R64-1-1 respectively) have evolved to have higher expressing amino acid combinations in positions 3, 4 and 5 (GFP score > 4) in comparison to non-biased distribution in library. Majority of the endogenous sequences however maintain median levels of GFP score (2 < GFP score <4).

Full western blots included in main and supplementary figures:

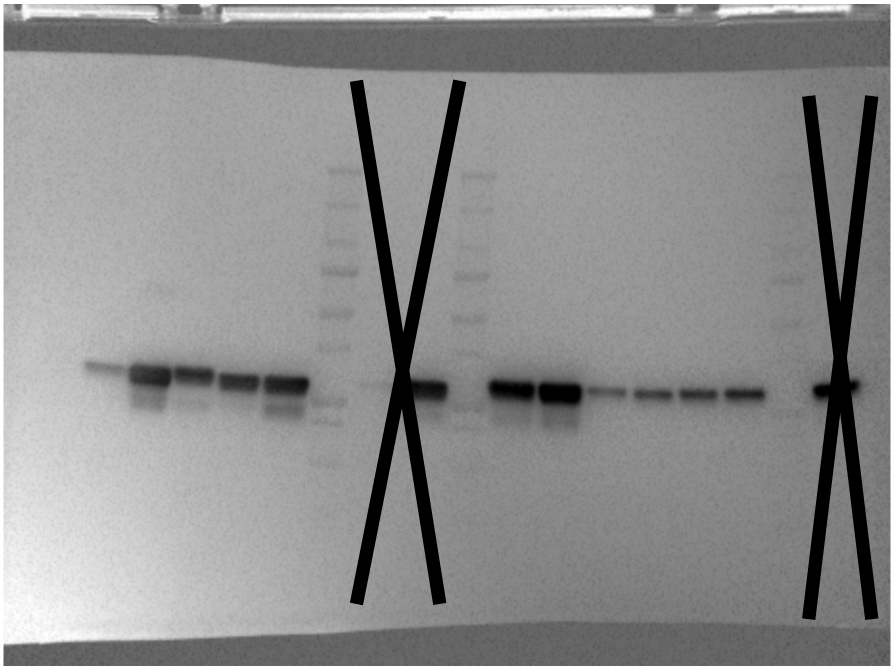

Supplementary Figure 25. Western blot used in Fig 4A and 5A

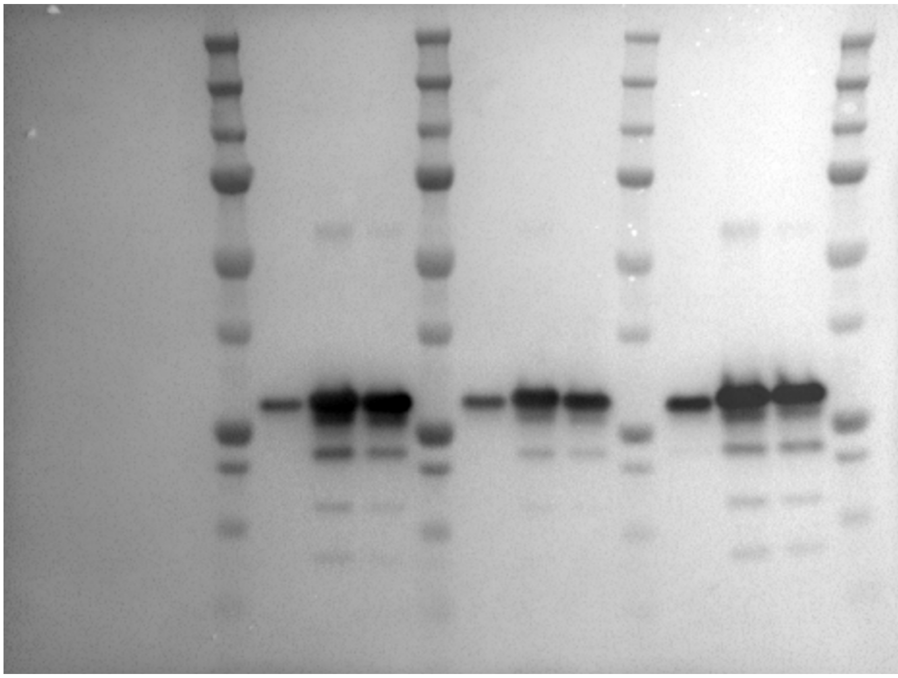

Supplementary Figure 26. Western blot used in Fig 4C

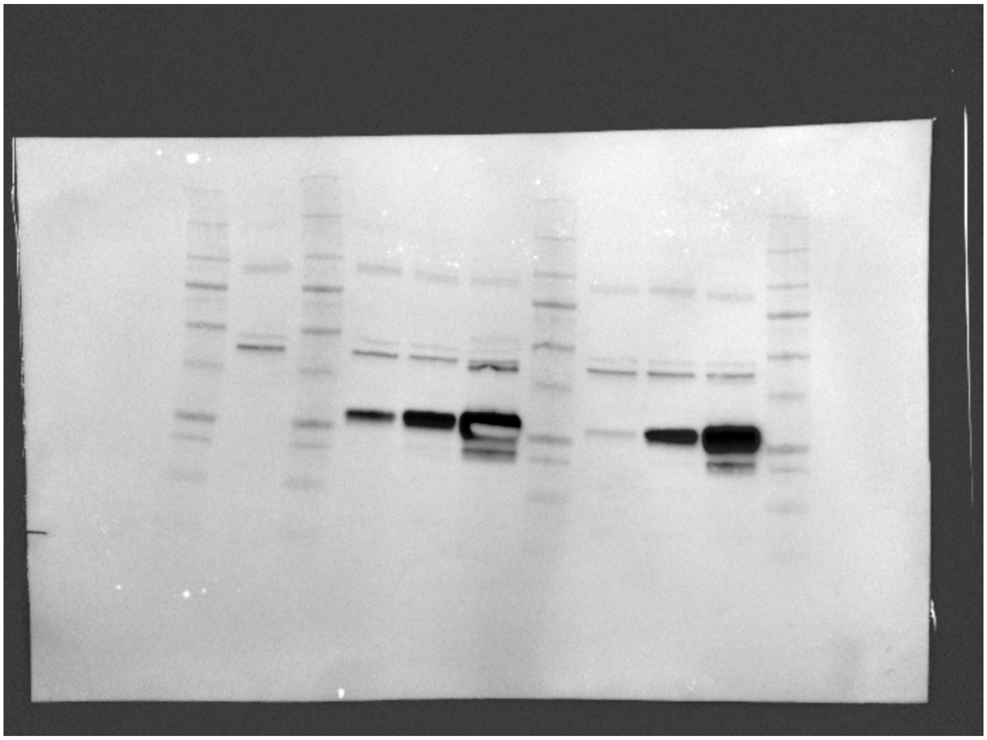

Supplementary Figure 27. Western blot used in Fig 4D

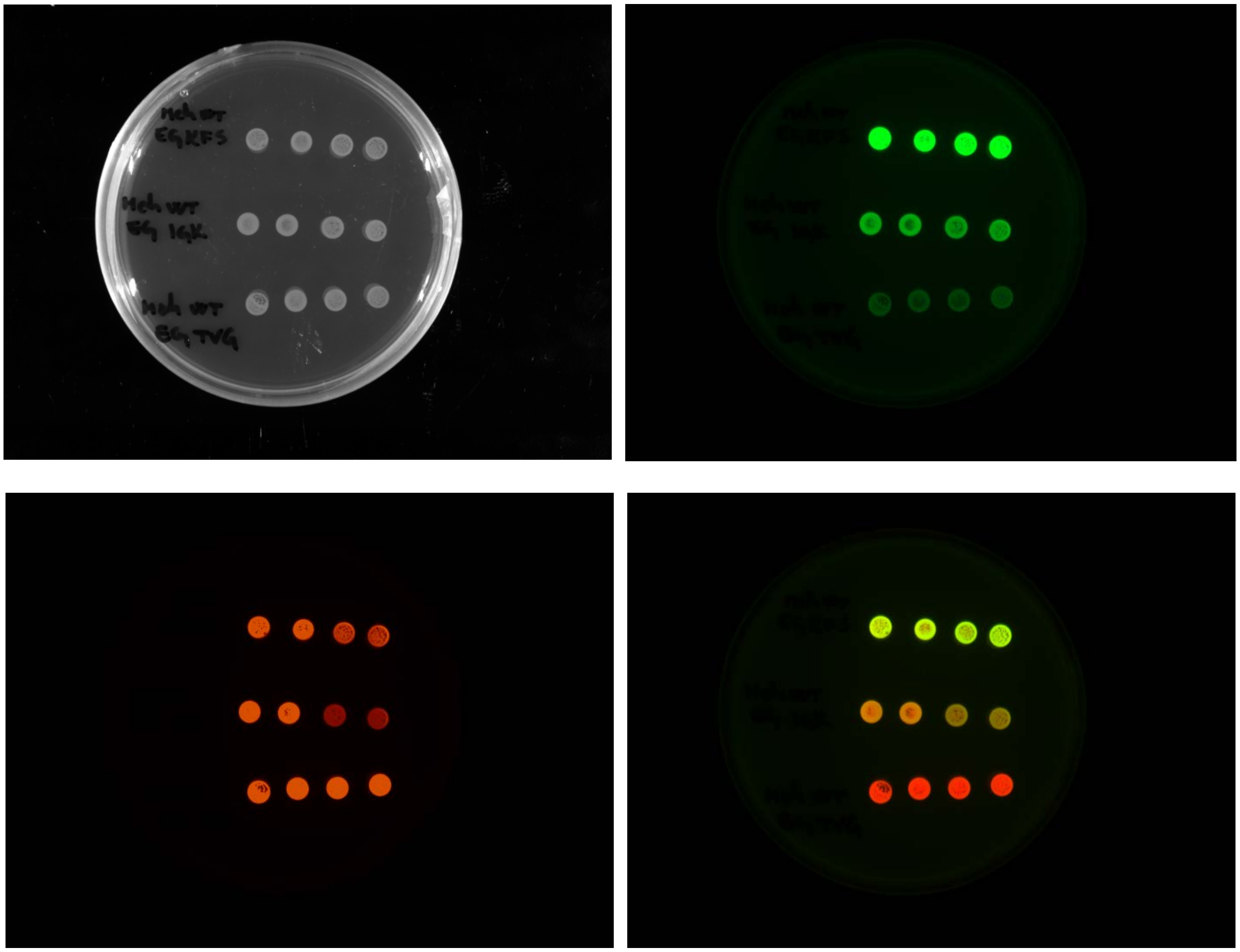

Supplementary Figure 28. Images used in Fig 5B

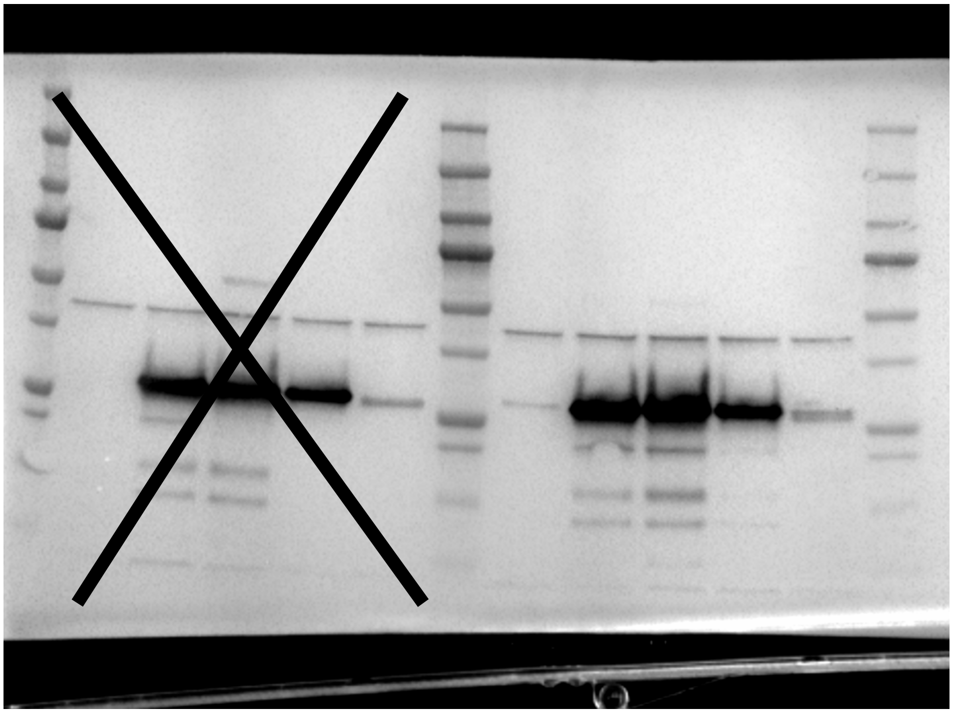

Supplementary Figure 29. Western blot used in Fig 5C

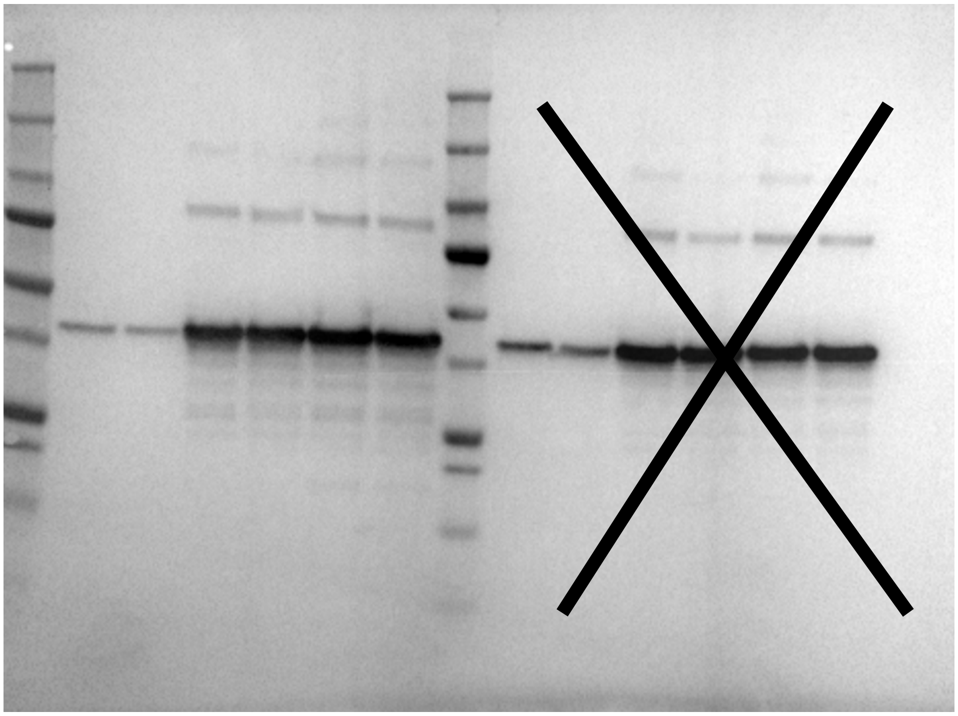

Supplementary Figure 30. Western blot used in Fig 5D

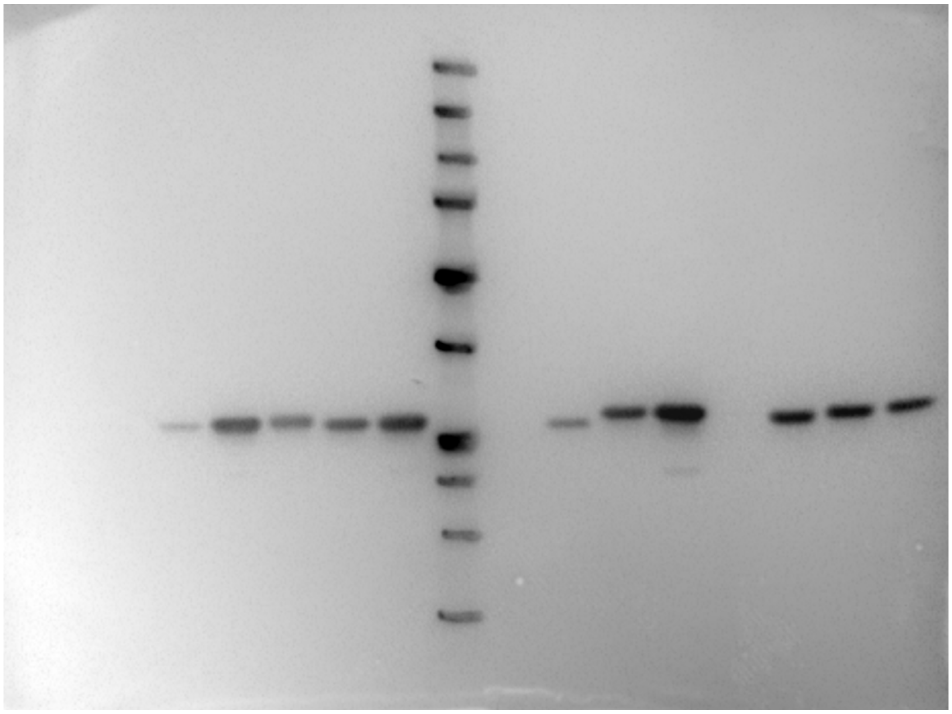

Supplementary Figure 31. Western blot used in Supplementary Fig 12 and 16

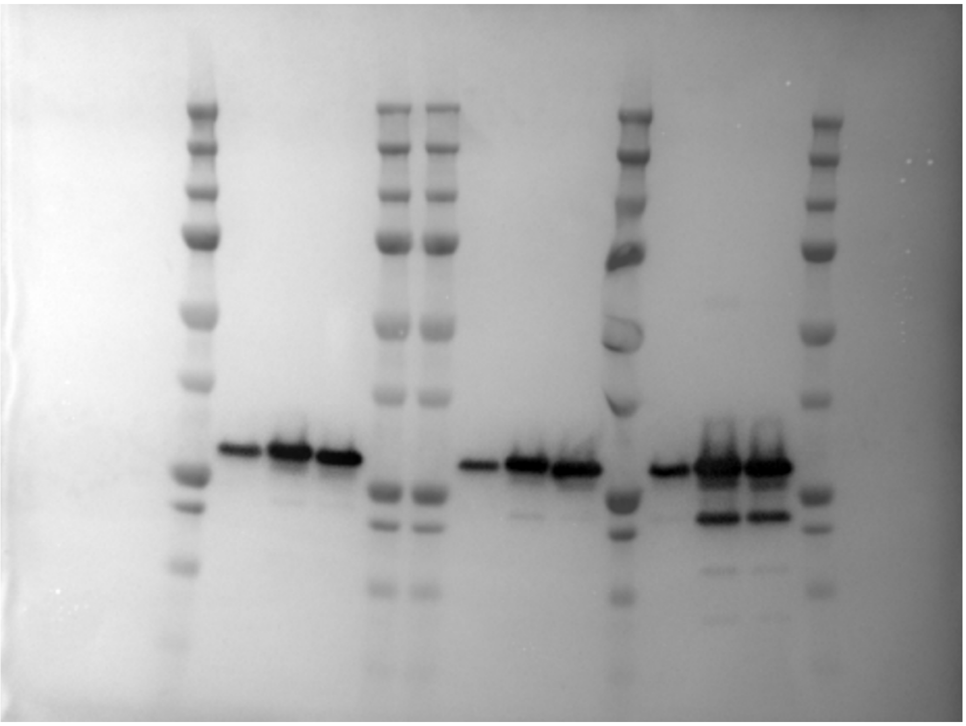

Supplementary Figure 32. Western blot used in Supplementary Fig 14

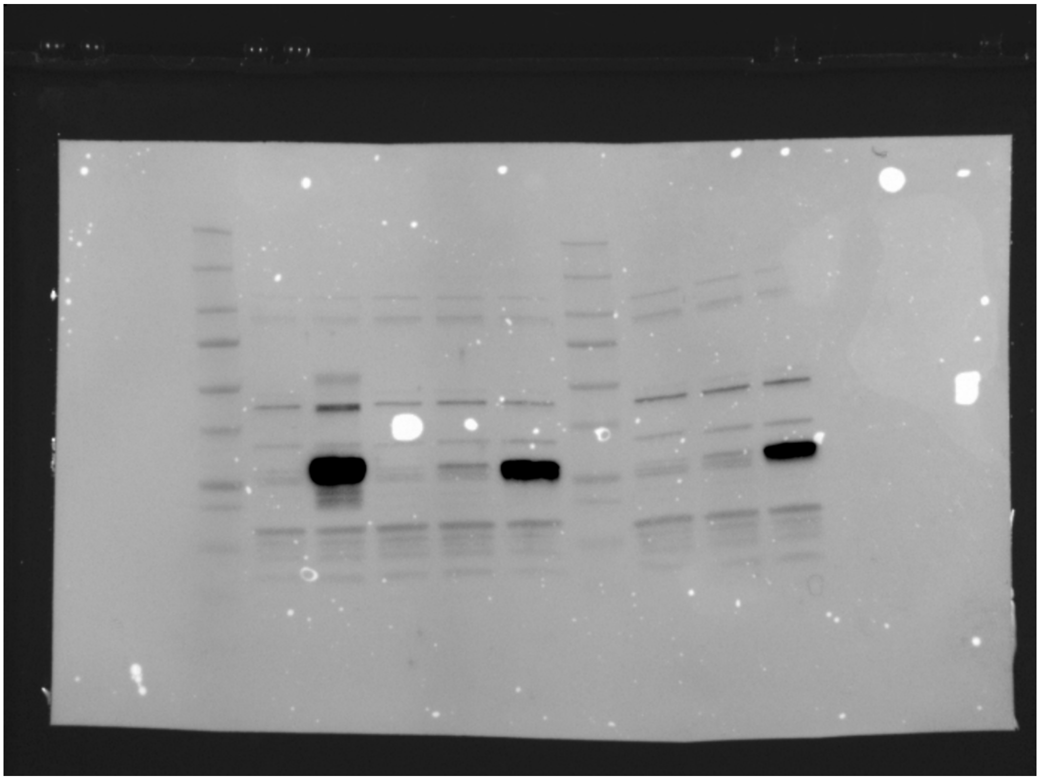

Supplementary Figure 33. Western blot used in Supplementary Fig 15

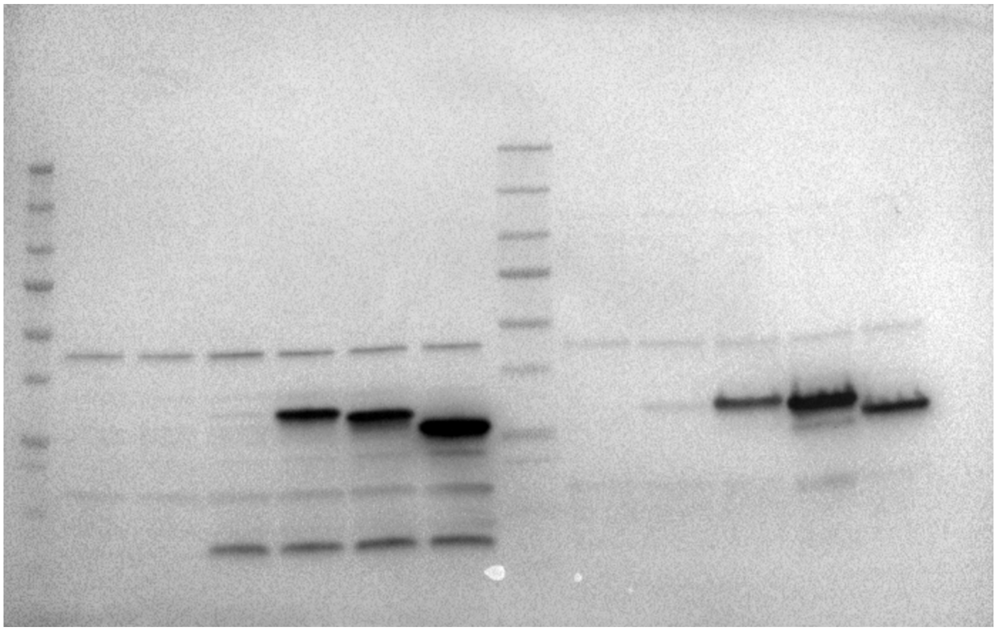

Supplementary Figure 34. Western blot used in Supplementary Fig 19
