# Supplementary material for: A short translational ramp determines the efficiency of protein synthesis
Source: Nat Commun. 2019 Dec 18;10:5774. doi: 10.1038/s41467-019-13810-1 (PMC6920384; doi:10.1038/s41467-019-13810-1)
Supplement: Supplementary file 2 — Reporting Summary [file 41467_2019_13810_MOESM2_ESM.pdf]

nature research

Corresponding author(s): Djuranovic Sergej

Last updated by author(s): Nov 21, 2019

Reporting Summary

Nature Research wishes to improve the reproducibility of the work that we publish. This form provides structure for consistency and transparency in reporting. For further information on Nature Research policies, see Authors & Referees and the Editorial Policy Checklist.

Statistics

For all statistical analyses, confirm that the following items are present in the figure legend, table legend, main text, or Methods section.

n/a	Confirmed	
☐	☒	The exact sample size (*n*) for each experimental group/condition, given as a discrete number and unit of measurement
☐	☒	A statement on whether measurements were taken from distinct samples or whether the same sample was measured repeatedly
☐	☒	The statistical test(s) used AND whether they are one- or two-sided<br>*Only common tests should be described solely by name; describe more complex techniques in the Methods section.*
☒	☐	A description of all covariates tested
☒	☐	A description of any assumptions or corrections, such as tests of normality and adjustment for multiple comparisons
☐	☒	A full description of the statistical parameters including central tendency (e.g. means) or other basic estimates (e.g. regression coefficient) AND variation (e.g. standard deviation) or associated estimates of uncertainty (e.g. confidence intervals)
☐	☒	For null hypothesis testing, the test statistic (e.g. *F*, *t*, *r*) with confidence intervals, effect sizes, degrees of freedom and *P* value noted<br>*Give P values as exact values whenever suitable.*
☐	☒	For Bayesian analysis, information on the choice of priors and Markov chain Monte Carlo settings
☒	☐	For hierarchical and complex designs, identification of the appropriate level for tests and full reporting of outcomes
☐	☒	Estimates of effect sizes (e.g. Cohen's *d*, Pearson's *r*), indicating how they were calculated

Our web collection on statistics for biologists contains articles on many of the points above.

Software and code

Policy information about availability of computer code

Data collection	MATLAB was used for analyses of smFRET data.
Data analysis	All scripts used for analysis are available at the Github repository under MIT license (https://github.com/cottrellka/EGFP_library_seq).<br>GraphPad Prism 7.0d, ImageQuant TL, Microsoft Excel 16.27

For manuscripts utilizing custom algorithms or software that are central to the research but not yet described in published literature, software must be made available to editors/reviewers. We strongly encourage code deposition in a community repository (e.g. GitHub). See the Nature Research guidelines for submitting code & software for further information.

Data

Policy information about availability of data

All manuscripts must include a data availability statement. This statement should provide the following information, where applicable:

- Accession codes, unique identifiers, or web links for publicly available datasets
- A list of figures that have associated raw data
- A description of any restrictions on data availability

All the data is included in manuscript. Sequencing data is deposited at http://www.ncbi.nlm.nih.gov/bioproject/590742.

Field-specific reporting

Please select the one below that is the best fit for your research. If you are not sure, read the appropriate sections before making your selection.

☒ Life sciences ☐ Behavioural & social sciences ☐ Ecological, evolutionary & environmental sciences

Life sciences study design

All studies must disclose on these points even when the disclosure is negative.

Sample size	These are indicated in the figure legends and/or methods.<br>For biochemical assays, three repeats were used and the fluorescence of each was measured in duplicates.<br>For library FACS sorting and sequencing biological duplicates were used.
Data exclusions	There was no data exclusion.
Replication	Biological replicates were included in analyses and submitted as separate files for sequencing. Western analysis was repeated on different days with different samples to ensure reproducibility.
Randomization	E. coli eGFP library randomization is based on primer and plasmid sequences. eGFP variants were created as defined in Methods file. Single clones are confirmed by selective plating and sequencing.
Blinding	Colony separation of E.coli cells with eGFP variants was done based on FACS gating protocol without previous knowledge of eGFP expression values.

Reporting for specific materials, systems and methods

We require information from authors about some types of materials, experimental systems and methods used in many studies. Here, indicate whether each material, system or method listed is relevant to your study. If you are not sure if a list item applies to your research, read the appropriate section before selecting a response.

Materials & experimental systems

n/a	Involved in the study	
☐	☒	Antibodies
☒	☐	Eukaryotic cell lines
☒	☐	Palaeontology
☒	☐	Animals and other organisms
☒	☐	Human research participants
☒	☐	Clinical data

Methods

n/a	Involved in the study	
☒	☐	ChIP-seq
☐	☒	Flow cytometry
☒	☐	MRI-based neuroimaging

Antibodies

Antibodies used	Primary antibodies for eGFP (JL-8; Clontech), penta-HIS (QIAGEN) or α-RF1 E.coli (Zaher Lab, Zaher and Green, Cell 2011) were used in all figures . Anti-mouse or anti-rabbit HRP conjugated antibodies (Cell Signaling) were used as secondary antibodies.
Validation	All antibodies were used for western analysis and sizes of the bands matched the predicted sizes of the proteins.<br>Antibodies for tags like 6xHis were validated by probing samples from E.coli that do not have tagged proteins. Secondary antibodies were validated using blots that were not probed with the appropriate primary antibody.

Flow Cytometry

Plots

Confirm that:

☒ The axis labels state the marker and fluorochrome used (e.g. CD4-FITC).

☒ The axis scales are clearly visible. Include numbers along axes only for bottom left plot of group (a 'group' is an analysis of identical markers).

☒ All plots are contour plots with outliers or pseudocolor plots.

☒ A numerical value for number of cells or percentage (with statistics) is provided.

Methodology

Sample preparation	Detailed protocol is described in Methods of the manuscript.
Instrument	Aria III flow cytometer (BD Biosciences).
Software	BD FACSDiva software.

Cell population abundance	Bin 1-4 each represent approximately 24% of the whole cell population depending on eGFP expression. Bin 5 represents 2.5% of the E.coli cells with highest eGFP expression based on relative fluorescence values (RFUs).
Gating strategy	The cells were sorted by level of eGFP expression into five bins with median eGFP fluorescence of 20, 120, 600, 3600, and 12,000 RFUs.

☐ Tick this box to confirm that a figure exemplifying the gating strategy is provided in the Supplementary Information.
