# Supplementary material for: A short translational ramp determines the efficiency of protein synthesis
Source: Nat Commun. 2019 Dec 18;10:5774. doi: 10.1038/s41467-019-13810-1 (PMC6920384; doi:10.1038/s41467-019-13810-1)
Supplement: Supplementary file 3 — Description of Additional Supplementary Files [file 41467_2019_13810_MOESM3_ESM.docx]

**Description of Additional Supplementary Files**

File name: Supplementary Data 1
Description: Raw Counts Experiment 1

File name: Supplementary Data 2
Description: Raw Counts Experiment 2

File name: Supplementary Data 3
Description: Annotated Counts Experiment 1

File name: Supplementary Data 4
Description: Annotated Counts Experiment 2

File name: Supplementary Data 5
Description: Heading Descriptions
